# Interruptin C, a Radioprotective Agent, Derived from *Cyclosorus terminans* Protect Normal Breast MCF-10A and Human Keratinocyte HaCaT Cells against Radiation-Induced Damage

**DOI:** 10.3390/molecules27103298

**Published:** 2022-05-20

**Authors:** Nipha Chumsuwan, Pasarat Khongkow, Sireewan Kaewsuwan, Kanyanatt Kanokwiroon

**Affiliations:** 1 Department of Biomedical Sciences and Biomedical Engineering, Faculty of Medicine, Prince of Songkla University, Hat Yai, Songkhla 90110, Thailand; noknipha@hotmail.com (N.C.); k.pasarat@gmail.com (P.K.); 2 Department of Radiology, Faculty of Medicine, Prince of Songkla University, Hat Yai, Songkhla 90110, Thailand; 3 Department of Pharmacognosy and Pharmaceutical Botany, Faculty of Pharmaceutical Sciences, Prince of Songkla University, Hat Yai, Songkhla 90110, Thailand; songsri.k@psu.ac.th; 4 Phytomedicine and Pharmaceutical Biotechnology Excellence Center, Faculty of Pharmaceutical Sciences, Prince of Songkla University, Hat Yai, Songkhla 90110, Thailand

**Keywords:** radioprotective agent, antioxidant, radiotherapy, breast cancer, DNA damage

## Abstract

Radiotherapy is a common method to treat cancers, with the goal of maximizing the dose to tumors while minimizing the dose to normal tissues. Radioprotectors can reduce the toxicity to normal tissues during radiotherapy. Several plant-derived compounds can function as radioprotectors by scavenging free radicals. We investigated the radioprotective activity of interruptin C from the fern *Cyclosorus terminans*. The molecular mechanism of interruptin C’s activity in X-ray-irradiated cells was evaluated. Superoxide dismutase activity was examined to investigate the antioxidant enzyme activity. Clonogenic cell survival was also investigated following radiation exposure. DNA damage and cell cycle progression were detected using micronuclei formation assays. DNA repair after irradiation was analyzed in a γH2AX assay. The levels of the proteins related to the radioprotective responses were analyzed by Western blotting. Interruptin C increased the antioxidant enzyme activity and significantly decreased the DNA damage by reducing the γH2AX foci and micronucleus formation in irradiated MCF-10A normal breast and HaCaT human keratinocyte cells. The apoptotic protein levels decreased, whereas the antiapoptotic protein levels increased. Interruptin C pretreatment increased the survival rate of irradiated MCF-10A and HaCaT cells. Moreover, the compound did not promote the survival of MDA-MB-231 and Hs578T breast cancer cells. Therefore, interruptin C may exert radioprotective activity without enhancing cancer cell proliferation.

## 1. Introduction

Many types of cancer treatment have been developed, such as surgery, chemotherapy, immunotherapy, and hormone therapy. Among the most common treatment for cancers is radiotherapy. In radiotherapy, ionizing radiations such as X-ray, gamma ray, or particles are used to shrink tumors and kill cancer cells. The goal of radiotherapy is to achieve the maximum tumor control while minimizing the toxicity to normal tissues [1]. Breast cancer is the most common cancer in women [2]. Thus, we initially focused on breast cancer treatment. Patients with breast cancer typically require radiotherapy, in addition to other treatments, such as surgery, chemotherapy, and hormone therapy. The need for radiotherapy depends on the types of surgery patients, tumor stage, and patient age and health. Ionizing radiation used in radiotherapy can damage not only tumors but also the surrounding normal tissues. The irradiation of normal tissues during radiotherapy can result in various complications, including early or late side effects such as moist desquamation, erythema, fibrosis, and atrophy [3].

Since 80% of the human body is composed of water, radiation damage can be caused by free radicals generated by the interaction of radiation with water [4]; these free radicals can react with macromolecules such as DNA, RNA, lipids, and proteins. Although advanced radiotherapy techniques such as intensity-modulated radiotherapy, volumetric arc radiotherapy, and image-guided radiotherapy can reduce the complication by reducing the volume of normal tissues exposed to radiation, these complications remain as major problems in patients undergoing radiotherapy. An alternative strategy for reducing the negative effects of radiotherapy is using radioprotectors, which are agents administered to protect against or reduce the toxicity of radiotherapy in normal tissues. These agents can be divided into three groups based on the timing of their delivery. First, some radioprotective agents or radioprotector are delivered before or at the time of irradiation. The second group is delivered at the time of irradiation or after irradiation but before the presentation of normal tissue toxicity. Finally, the third group is delivered to improve the normal tissue [5,6]. Radioprotective activity pathways are shown in Figure S1 in Supplementary Materials.

The first commercial radioprotector approved by FDA was the synthetic prodrug amifostine (also known as WR 2721). Amifostine is rapidly dephosphorylated at its thiol group by alkaline phosphatase, a membrane-bound enzyme present in both normal and tumor tissues to generate the active form of the prodrug (WR-1065). The concentration of alkaline phosphatase is higher in normal cells than in most types of tumor cells; therefore, the conversion of WR-2721 to WR 1065 is more effective in normal tissues. Additionally, most type of cancers show poor vascularization compared with normal tissues, resulting in a low supply of drugs to cancer cells. The main mechanism of WR 1065 is to scavenge reactive species using the SH group of the molecule, thus preventing DNA damage [7]. However, amifostine is not commonly used in the clinical routine because of its high cost and side effects such as nausea, vomiting, and hypotension [8]. These effects can lead to treatment interruption, resulting in the significant loss of local tumor control, particularly in rapid repopulating tumors such as head and neck cancer [9].

**Figure 1:**
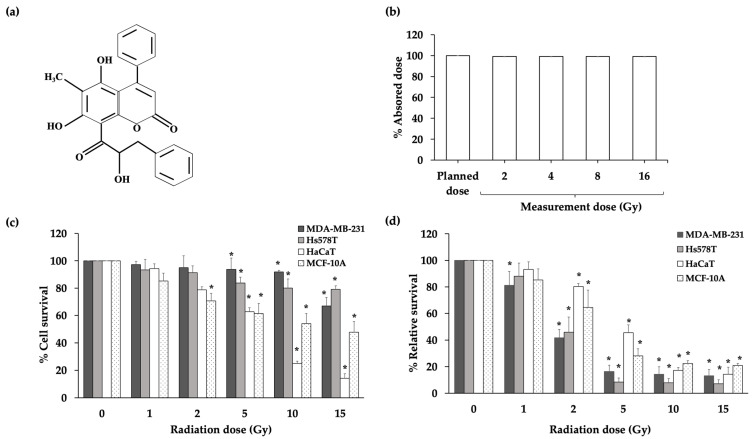
(**a**) The chemical structure of interruptin C compound. (**b**) Absorbed amount of planned dose and measured dose. Data represent the mean ± SD values from three independent experiments. The significance of the mean differences was analyzed using a paired *t*-test. * *p* < 0.05 compared to the planned dose. (**c**) Percentage of cell survival in the MTT assay at 72 h after irradiation of the cancer cell lines (MDA-MB-231 and Hs578T) and normal (HaCaT and MCF-10A) cell lines. Data represent the mean ± SD values from three independent experiments. The significance of the mean differences was analyzed using Kruskal–Wallis with Dunn’s test. * *p* < 0.05 compared to the nonirradiated cells. (**d**) Long-term cell survival in the clonogenic cell survival assay of irradiated cancer (MDA-MB-231 and Hs578T) and normal (HaCaT and MCF-10A) cell lines. Data represent the mean ± SD values from three independent experiments. The significance of the mean differences was analyzed using Kruskal–Wallis with Dunn’s test. * *p* < 0.05 compared to the nonirradiated group.

Therefore, safe and natural radioprotectors are needed. Natural compounds with free radical scavenging activity were shown to protect against the main radiation damage caused by free radicals and show potential for use as radioprotectors to reduce the effect of high-energy X-ray irradiation on normal cells [10,11]. The fern *Cyclosorus terminans* (Panigrahi) Panigrahi, *Thelypteris terminans* Panigrahi, Phytologia, *Amphineuron terminans* (Hook.) Holttum of the family Thelypteridaceae has long been consumed in Northern Thailand [12,13,14,15]. The major bioactive components of *C. terminans* extracts are coumarins composed of interruptin A, B, and C. Previous studies demonstrated the anticancer, antibacterial, and potent free radical scavenging activity of interruptins [16,17]. We selected interruptin C derived from *C. terminans* to investigate its radioprotective potential and the molecular mechanism of its radiation response in X-ray-irradiated normal cells. The chemical structure of interruptin C compound (C_25_H_20_O_6_) is shown in Figure 1a. This compound may be further developed as a radioprotective agent to minimize the complications in the normal tissues of patients undergoing radiotherapy.

## 2. Results

### 2.1. Interruptin C Showed Free Radical Scavenging Activity and Low Toxicity

First, the irradiation setup for the cell culture was optimized. The measured absorbed dose was compared with the planned dose. The difference between the measured absorbed dose and planned dose was less than 1%. Thus, this condition was used for further experiments (Figure 1b). To optimize the radiation dose, the cell viability was investigated at 72 h after irradiation using an MTT assay and clonogenic cell survival assay (Figure 1c,d). The results of the long-term survival and clonogenic cell survival assay showed that 2-Gy radiation damages more than 50% of cancer cells and 80% of normal cells. Therefore, a radiation dose of 2 Gy was used for further study.

**Figure 2:**
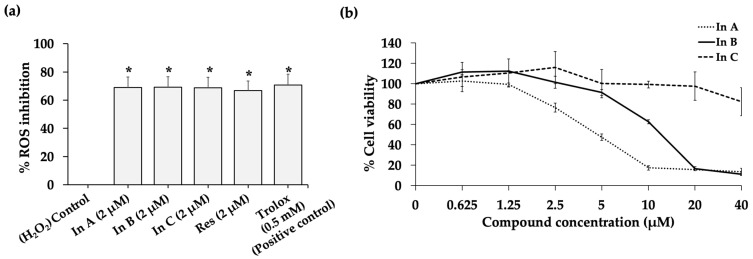
(**a**) Reactive oxygen species (ROS) scavenging activity was measured by the DCFH-DA assay after 2 µM In A (interruptin A), 2 µM In B (interruptin B), 2 µM In C (interruptin C), and 2 µM Res (resveratrol) compound treatments. A small amount (0.5 mM) of Trolox was used as a positive control. Data represent the mean ± SD values from three independent experiments. The significance of the mean differences was analyzed using Kruskal–Wallis with Dunn’s test. * *p* < 0.05 compared to the control. (**b**) Cell viability in the MTT assay at 72 h after treatment with In A, In B, and In C in HaCaT human keratinocytes. Data represent the mean ± SD values from three independent experiments.

We determined the free radical scavenging activity of interruptins in a DCFH-DA assay. The cells were treated with H_2_O_2_ to generate oxidative stress. DCFH was oxidized by reactive oxygen species (ROS) into fluorescent DCF with a high fluorescence intensity, indicating oxidative stress. The capacity for scavenging ROS was determined as %ROS inhibition. Interruptins A, B, and C showed high antioxidant activity with 68.77–69.19% inhibition, which was similar to the positive control, Trolox (Figure 2a). Cell viability following treatment with interruptins A, B, and C was also assessed using the HaCaT cell line to select the appropriate concentration of interruptin. The treatment with 2 µM of each interruptin compound led to a high cell viability of more than 80% (Figure 2b). Although interruptins A, B, and C showed high ROS scavenging activities, interruptin C showed the lowest cytotoxicity. Thus, the radioprotective activity of 2 µM interruptin C was further investigated.

### 2.2. Interruptins Increased Survival of Normal Cells and Did Not Affect Proliferation of Cancer Cells

**Figure 3:**
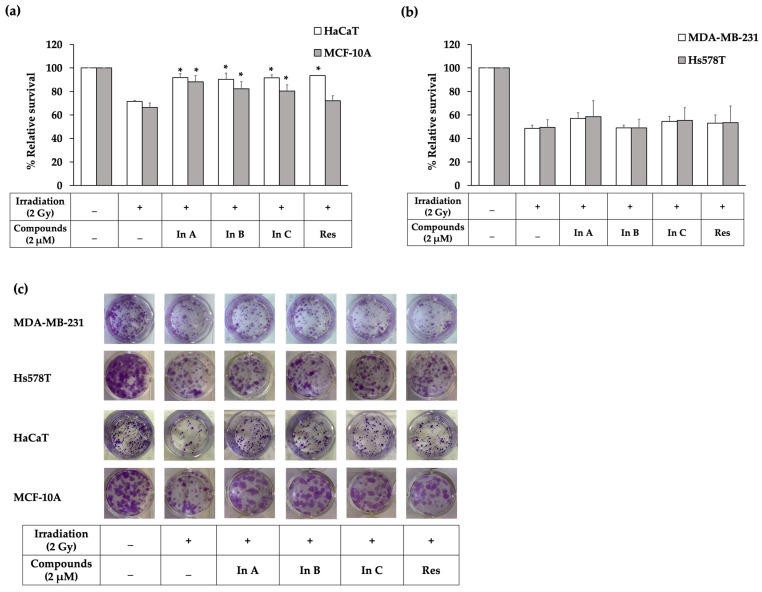
(**a**) Relative survival in the clonogenic cell survival assay after treatment with In A (interruptin A), In B (interruptin B), In C (interruptin C), and Res (resveratrol) in HaCaT and MCF-10A normal cells. Data represent the mean ± SD values from three independent experiments. The significance of the mean differences was analyzed using Kruskal–Wallis with Dunn’s test. * *p* < 0.05 compared to the irradiation-alone group. (**b**) Relative survival in the clonogenic cell survival assay after treatment with In A, In B, In C, and Res in HaCaT and MCF-10A normal cells. Data represent the mean ± SD values from three independent experiments. The significance of the mean differences was analyzed using Kruskal–Wallis with Dunn’s test. * *p* < 0.05 compared to the irradiation-alone group. (**c**) Images from the clonogenic cell survival assay after treatment with In A, In B, In C, and Res in MDA-MB-231 and Hs578T breast cancer cells and HaCaT keratinocyte and MCF-10A normal breast cells.

Next, we explored the radioprotective effect of interruptins A, B, and C in normal and cancer cells in a clonogenic cell survival assay. For HaCaT skin keratinocytes, pretreatment with interruptins maintained the cell survival at 91.63%, whereas the survival of irradiated cells was 71.38%. Pretreatment with interruptins increased the cell survival from 66.41% in the irradiation alone group to 80.32% in the MCF-10A normal breast cell group (Figure 3a). For MDA-MB 231 and Hs578T breast cancer cells, the survival in the cell irradiation alone group and compound pretreatment group were 48.53–54.5% and 49.3–55.26%, respectively (Figure 3b). Images of the clonogenic cell survival assay in the MDA-MB-231, Hs578T, HaCaT, and MCF-10A cell lines are shown in Figure 3c and indicate that interruptins can protect normal cells from radiotherapy and do not promote the proliferation of breast cancer cells.

### 2.3. Oxidative Stress from Radiation Exposure Was Reduced by Pretreatment with Interruptin C

**Figure 4:**
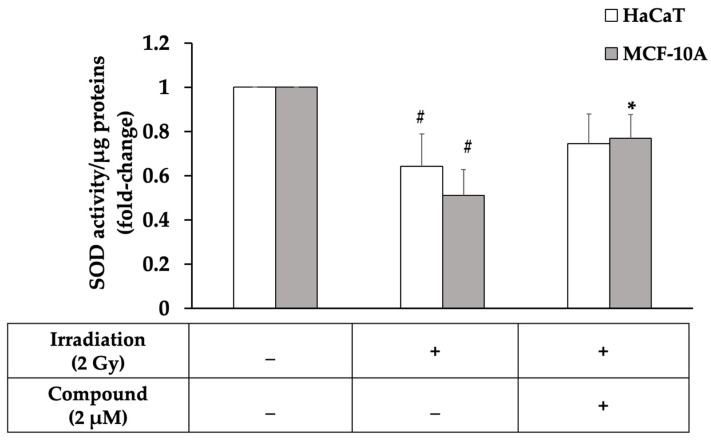
Superoxide dismutase (SOD) activity in normal cells and HaCaT and MCF-10A cell lines treated with interruptin C derived from *C. terminans* before 2-Gy irradiation. Data represent the mean ± SD from three independent experiments. The significance of the mean differences was analyzed using Kruskal–Wallis with Dunn’s test. * *p* < 0.05 compared to the irradiation-alone group. # *p* < 0.05 compared to the nonirradiated group.

Since interruptin C showed the lowest cytotoxicity and similar radioprotective activity among all the compounds, interruptin C was selected to analyze its molecular mechanism. The cellular antioxidant activity of interruptin C following the pretreatment of HaCaT and MCF-10A-irradiated cells was analyzed by measuring the superoxide dismutase (SOD) activity (Figure 4). A 2-Gy irradiation dose lowered the SOD activity of irradiated cells compared to in nonirradiated cells. Moreover, interruptin C pretreatment enhanced the SOD activity, particularly in MCF-10A cells, suggesting that interruptin C has potent antioxidant activity.

### 2.4. Interruptin C Decreased DNA Damage after Irradiation

**Figure 5:**
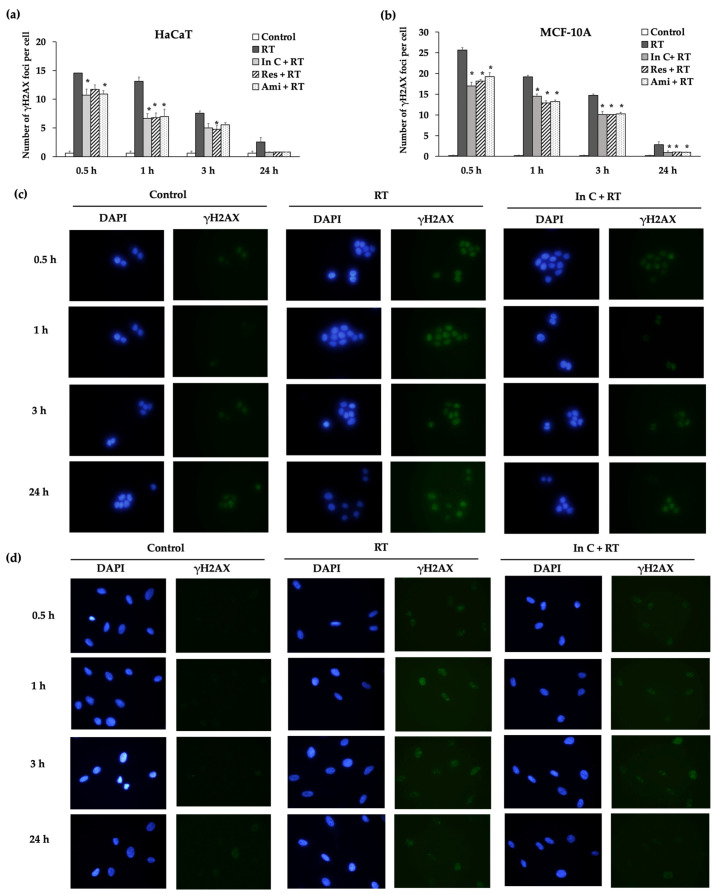
(**a**) γH2AX assay to detect the number of γH2AX foci per cell at 0.5, 1, 3, and 24 h after irradiation. The cells were pretreated with In C (interruptin C), Res (resveratrol), and Ami (amifostine) in HaCaT and (**b**) MCF-10A cell lines. The cells were nonirradiated (Control), irradiated alone (RT), and treated with interruptin C before 2-Gy cell irradiation (In C+RT). Data represent the mean ± SD values from three independent experiments. The significance of the mean differences was analyzed using Kruskal–Wallis with Dunn’s test. * *p* < 0.05 compared to the irradiation-alone group. (**c**) HaCaT and (**d**) MCF-10A cells after irradiation in the γH2AX assay with the γH2AX foci and DAPI staining at each time point.

We investigated whether interruptin C can decrease DNA damage and enhance DNA repair. The formation of γH2AX molecules corresponds to DNA double-stranded breaks (DSBs) and can be conveniently evaluated in assays. In this study, the irradiation of HaCaT and MCF-10A cells at 2 Gy induced larger numbers of DNA DSBs with a high γH2AX foci compared to the nonirradiated (control) group. However, interruptin C pretreatment significantly reduced the DNA DSBs (Figure 5a,b). Images of normal cells after irradiation in the γH2AX assay at each time point are shown in Figure 5c,d. Interruptin C, resveratrol, and amifostine showed same capacity for reducing DNA DSBs.

**Figure 6:**
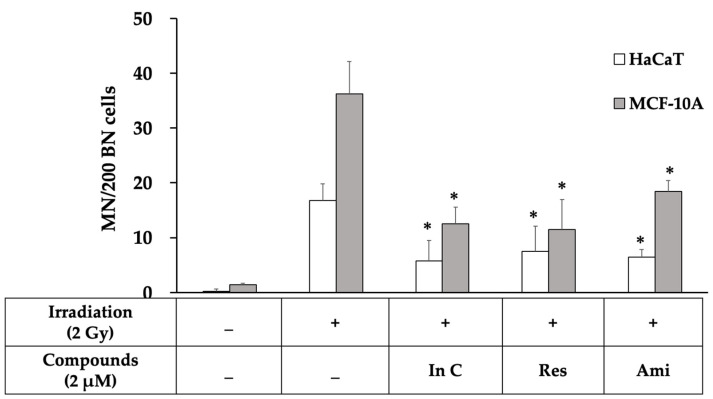
The total number of micronuclei in 200 binucleated cells from the micronuclei formation assay in HaCaT and MCF-10A cell lines treated with In C (interruptin C), Res (resveratrol), and Ami (amifostine) before 2-Gy irradiation. Data represent the mean ± SD values from three independent experiments. The significance of the mean differences was analyzed using Kruskal–Wallis with Dunn’s test. * *p* < 0.05 compared to the irradiation-alone group.

To confirm whether interruptin C decreases the radiation-induced DNA damage, a micronuclei formation assay was performed. Micronuclei, a biomarker of chromosome breakage or chromosome loss, were scored after blocking cytokinesis with the agent cytochalasin B. The number of micronuclei (MN) was counted from 200 binucleated cells. Three independent experiments showed that 2-Gy irradiation increased DNA damage by increasing the micronuclei formation by more than in the control group (Figure 6). The treatment of the cells with the compounds before irradiation significantly decreased the MN formation compared with cell irradiation alone. The interruptin C pretreatment decreased the number of γH2AX foci and MN formation, indicating reduced DNA damage in irradiated cells. Interruptin C showed similar activity as resveratrol and amifostine in HaCaT (Figure 5a) and MCF-10A cells (Figure 5b).

### 2.5. Effect of Interruptin C on DNA Damage Response in Irradiated Normal Cells

**Figure 7:**
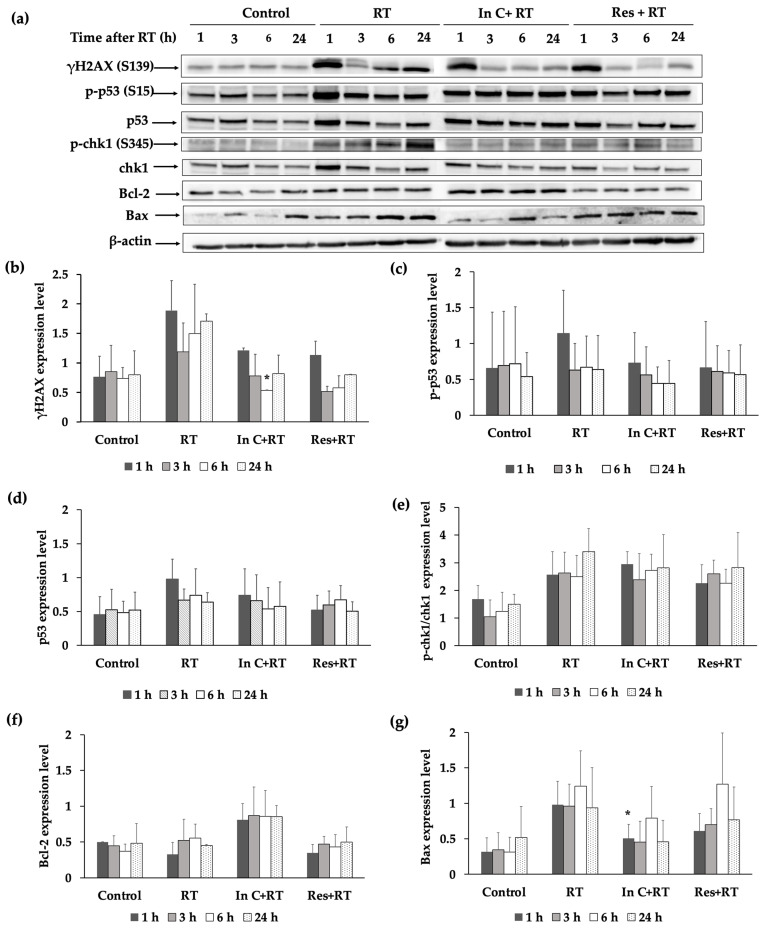
(**a**) Representative protein bands are shown under different conditions, including cells without irradiation (control), irradiated cells (irradiation), interruptin C treatment prior to irradiation (In C+RT), and resveratrol treatment prior to irradiation (Res+RT) at various incubation times after the irradiation exposure of HaCaT cells. The protein expression of (**b**) γH2AX (S139), (**c**) p-p53 (S15), (**d**) p53, (**e**) p-Chk1/Chk1 (S345), (**f**) Bcl-2, and (**g**) Bax was quantified. The values were normalized to β-actin intensity. Data represent the mean ± SD values from three independent experiments. The significance of the mean differences was analyzed using Kruskal–Wallis with Dunn’s test. * *p* < 0.05 compared to the irradiation-alone group.

We identified the radioprotective activity of interruptin C in HaCaT keratinocytes. To confirm the results, molecules related with the radioprotective effect were investigated by performing a Western blot analysis. In HaCaT cells treated with RT alone, γH2AX (Figure 7a,b) (Figure S5 in Supplementary Materials) and Bax pro-apoptosis protein (Figure 7a,g) (Figure S9 in Supplementary Materials) were highly expressed at 6–24 h after radiation. Radiation also enhanced the expression levels of p-p53 (S15) (Figure 7c) (Figure S6 in Supplementary Materials) and p-chk1/chk1 (Figure 7e) (Figure S7 in Supplementary Materials). However, interruptin C insignificantly reduced the levels of p-p53 (S15) and p-chk1/chk1 at 1 and 3 h after irradiation, respectively (Figure 7a,c,e). The interruptin C-treated cells showed the lowest γH2AX level at 6 h after irradiation (Figure 7b). The anti-apoptosis protein Bcl-2 was highly expressed in interruptin C-pretreated cells at all time points compared to in the other groups (Figure 7f) (Figure S8 in Supplementary Materials). These results suggest that interruptin C can reduce DNA damage and apoptotic proteins and increase antiapoptotic proteins.

## 3. Discussion

Radiotherapy is among the most common methods used for cancer treatment. This curative or palliative approach is used in more than 80% of patients with cancer [4]. A balance between the dose to the tumor and threshold dose to the surrounding normal tissues is required to obtain optimal results. Normal tissues should be protected from radiation-induced damage. Thus, radioprotectors play crucial roles in radiotherapy.

Ionizing radiations cause damage to cells, tissues, and organs through a sequence of molecular events. The main radiation-induced damage is caused by free radicals, which are formed by the interaction of radiation with water, because cells or tissues contain more than 80% water. These free radicals are very reactive and can damage macromolecules within the cell, such as DNA, RNA, lipid, protein, and the cell membrane [18], by irreparably disrupting their structures. These changes lead to apoptosis, necrosis, mitotic death, autophagy, and senescence. Hence, radioprotectors with free radical scavenging activity that reduce the number of free radicals must be developed. Previous studies have suggested that the number of hydroxyl groups on the ring structure of coumarin is correlated with free radical scavenging activity; therefore, we predicted that hydroxyl groups on the structure of interruptin C are related to free radical scavenging activity, leading to decreased radiation-induced damage.

Mirzayans et al. [19] reported that the 50% inhibitory doses for the breast cancer cell line MCF7 were 4 and 2 Gy in the MTT assay and clonogenic cell survival assay, respectively. In our study, cell viability and cell survival assays showed that radiation damaged more than 60% of the cancer cells at a radiation dose of 2 Gy and 40% of normal cells. Therefore, we used a radiation dose of 2 Gy for further study. The treatment of normal cells with the interruptin compounds showed that 2 µM maintained the cell viability at more than 80%; therefore, we used this dose to study the radioprotective activity of compounds.

Exposure to ionizing radiation produces free radicals within cells. Antioxidant enzymes, including MnSOD, Cu/ZnSOD, and glutathione peroxidase, are key intracellular scavengers. Sun et al. [20] showed that, among MnSOD, Cu/ZnSOD, and glutathione peroxidase, MnSOD plays the most important role in protecting cells from ROS injury during radiation exposure. SOD activity was reduced under oxidative stress conditions, and radioprotection was required to enhance SOD activity. Candas et al. [21] suggested that cyclinB1/Cdk1 regulates MnSOD through Ser106 phosphorylation, resulting in increased MnSOD activity, along with increased mitochondria function and resistance to radiation-induced apoptosis. We found that 2-Gy irradiation significantly decreased SOD activity. In MCF-10A cells, interruptin pretreatment significantly increased the SOD activity. Although HaCaT cells pretreated with interruptin showed a higher SOD activity compared to the cells treated with irradiation alone, the difference was not significant. Previous research demonstrated that HaCaT cells exhibited lower levels of intracellular SOD enzymes than other cell lines. Additionally, after exposure to stress such as ionizing radiation and ultraviolet rays, the level of the SOD enzyme was significantly reduced. It was thought to be mediated through inflammatory cytokines such as IL-1α and TNF-α [22,23,24]. In MCF-10A, it showed higher intracellular levels of SOD enzymes than other cell lines [25,26]. In this study, we found that a 2-Gy irradiation dose significantly decreased the SOD activity of irradiated cells when compared to nonirradiated cells. Furthermore, interruptin C pretreatment enhanced the SOD activity in both cell lines, although it was still insignificantly different in HaCaT.

The inability to proliferate infinitely and loss of reproductive integrity are common factors used to describe cell death [27]. Interruptin C pretreatment increased the cell survival and increased the number of colonies after irradiation. For normal HaCaT and MCF-10A cells, the number of colonies was significantly increased after treatment with interruptins compared to cells treated with radiation alone. Interruptin C can protect cells from radiotherapy equally to resveratrol, a positive control. The MDA-MB-231 and Hs578T breast cancer cells showed an insignificant increase in colonies after treating the cells with interruptins compared to cells treated with radiation alone. Thus, interruptin did not promote the proliferation of breast cancer cells.

The phosphorylation of the histone H2AX (γH2AX) foci indicates the presence of DNA DSBs, whereas the disappearance of the foci is correlated with DNA damage repair. γH2AX foci are often used as a marker for monitoring the DNA damage and repair dynamics of cells after radiation exposure. DNA DSBs are the major mode of radiation damage. After radiation exposure, γH2AX foci are rapidly formed, reaching their maximum numbers between 30 and 60 min. Therefore, a 1-h post-irradiation time point was considered as the initial time point of DNA DSB formation. The γH2AX foci were lost because of DNA repair. Typically, time points of 4–24 h post-irradiation are used to monitor DNA repair. The γH2AX assay can be used to evaluate the clinical response of DNA-targeted therapies such as chemotherapy and radiotherapy. We investigated the DNA damage and repair dynamics after irradiation with the interruptin pretreatment. At 2-Gy irradiation, the DNA DSBs were increased and γH2AX foci levels were high compared to in non-irradiated cells. However, interruptin C pretreatment decreased DNA DSBs and reduced the γH2AX foci. The change in γH2AX foci indicated that DNA damage and repair occurred after radiation exposure [28,29,30]. Mariotti et al. [31] demonstrated that the γH2AX assay can be used to evaluate DNA DSB induction and repair after single acute radiation exposure. The induction of γH2AX foci was affected by the initial radiation exposure, with a smaller number of foci induced by subsequent radiation exposure. The full recovery time of γH2AX foci induction was quantified for 12 h, and the 1:1 relationship between radiation-induced DNA DSBs and foci counts was investigated under multiple irradiation scenarios. Interruptin C decreased the DNA damage and increased the DNA repair.

For the radiation response, which occurs when the energy of ionizing radiation is absorbed by the cell, there are many radiation-induced damage mechanisms, including free radical production, inflammatory activation, and vascular endothelial dysfunction. Through the indirect action of X-rays and gamma rays, free radicals are generated. Free radicals could break chemical bonds, resulting in chemical changes. A radiation response can occur, including DNA SSB, DNA DSBs, and DNA–protein crosslinks. Multiple pathways are involved in genomic maintenance after exposure to radiation [32,33]. Several studies were performed suggesting that the DNA repair of cancer cells is slower than normal cells. Cancer cells contain several molecular characteristics, including inactivation of the BRCA, a high rate of p53 mutations, and activation of the PI3K pathways. It contributes to DNA repair deficiencies and genomic instability [34,35,36,37,38]. Furthermore, various proteins in cell damage pathways increase the radiosensitivity of the fast-doubling cancer cells Although the compounds scavenge free radicals produced the by interaction of radiation with water within the cells, other mechanisms of response, such as the inflammatory pathway, may occur [39,40,41]. The presence of γH2AX at early time points indicates DNA damage. Even though we did not investigate oxidative stress and DNA damage in cancer cells in this study, the clonogenic assay used as a long-term survival method to confirm that compound’s increased cell survival of normal cells did not affect the survival of cancer cells.

Cytokinesis-blocked MN or MN formation is one form of radiation-induced damage. MN formation occurred post-mitosis and was scored in binucleated cells posttreatment with cytochalasin B [42,43]. The activity of radioprotectors was assessed in a cytokinesis-blocked MN formation assay. Cheki et al. [43] reported that radioprotector treatment without irradiation did not increase the number of MN and showed no cytostatic effect in human lymphocytes. In addition, radioprotector treatment prior to irradiation significantly reduced radiation-induced apoptosis in human lymphocytes. We evaluated the long-term DNA damage repair by performing an MN formation assay. Cells treated with interruptin C before irradiation significantly decreased the DNA damage, as indicated by a lower MN formation compared with cell irradiation alone. Thus, interruptin C can decrease DNA damage and exert radioprotective activity.

In addition, the protein-related radioprotective response was examined. Radiation enhances the expression levels of p-p53 (S15) and p-chk1/chk1 in HaCaT cells. However, interruptin C lowered the levels of these proteins, particularly at 1 h after irradiation. Interruptin C also reduced DNA damage by decreasing the level of γH2AX, specifically at 3 h after irradiation. Interruptin C pretreatment protected HaCaT cells from radiation by increasing the level of the antiapoptotic protein Bcl-2 and reducing the level of the pro-apoptotic protein Bax. Therapies taking advantage of the defects in DNA repair pathways have been explored in breast cancer, ovarian cancer, and other tumor types.

**Figure 8:**
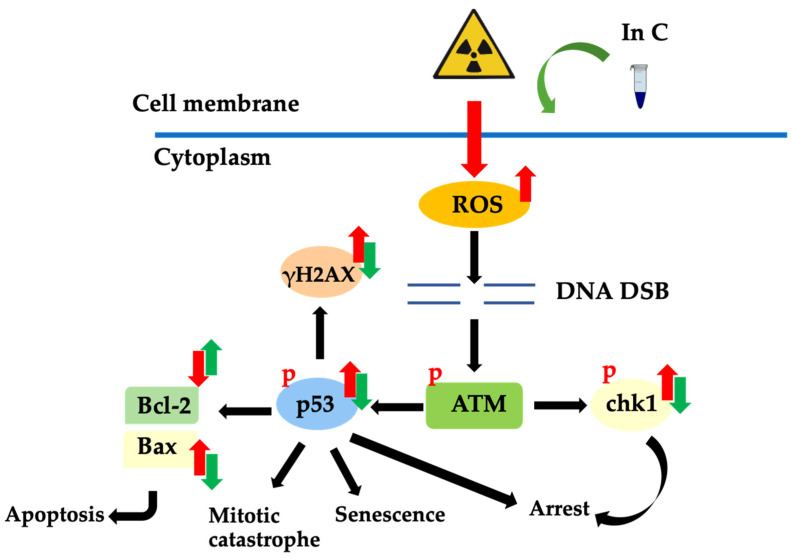
Downstream molecules and effects following DNA damage due to ionizing radiation. Reactive oxygen species (ROS) generated after irradiation can disrupt macromolecules, including DNA. This process activates ataxia–telangiectasia-mutated (ATM) and downstream molecules such as p53 and chk1. γH2AX molecules are key in the response to DNA double-stranded breaks (DSB). When cell death occurs via apoptosis, pro-apoptosis proteins are increased, and anti-apoptosis proteins are decreased. Interruptin C can protect cells from radiation by decreasing DNA damage, enhancing DNA repair, inducing anti-apoptosis proteins, and reducing pro-apoptosis proteins.

Radiation-induced damage is mainly caused by free radicals, and DSBs are the most common form of DNA damage associated with ionizing radiation. The occurrence of DSBs is followed by a process involving the activation of p53 and induction of cell cycle arrest. Irreparable damage leads to cell death, including apoptosis, necrosis, mitotic catastrophe, and senescence. Finally, tissue damage or complications can occur. Interruptin C functions as a radioprotector and reduces the formation of free radicals, induces DNA repair, and activates the cellular protective enzyme SOD. Furthermore, interruptin C can reduce the levels of pro-apoptosis proteins and increase the levels of anti-apoptosis proteins (Figure 8).

Several strategies can be used to develop radioprotectors. We focused on the scavenging of free radicals as the main mechanism of action, as radiation-induced damage is mediated by free radical formation. DNA can be protected by reducing the DNA damage caused by radical scavenging, followed by increasing DNA repair to improve radioprotection and recovery. Thus, natural compounds with free radical scavenging activity show potential as radioprotectors. Many studies have identified compounds with radioprotective activity using in vitro, ex vivo, and in vivo models [44,45,46,47,48] but, also, observed inflammasome activation in many immune cell types following radiation exposure. Radioprotectors may prevent radiation-induced inflammasome activation, although the mechanism requires further analysis. The interruptin compound derived from the fern *C. terminans* shows anticancer and antibacterial activities. Radioprotective activity was observed at a low interruptin concentration of 2 µM compared with 0.5 mM of vanillin from a vanilla orchid and 2.2 µM of resveratrol from grapes [11,40]. Although various agents have been shown to exert radioprotective effects in laboratory studies, most have failed before reaching the preclinical stage because of their toxicity and side effects. Interruptin compounds show promising radioprotective activity and potential for development as radioprotectors.

## 4. Materials and Methods

### 4.1. Compound Preparation

For natural compound preparation, interruptins A, B, and C were provided by Associate Professor Dr. Sireewan Kaewsuwan, Department of Pharmacognosy and Pharmaceutical Botany, Faculty of Pharmaceutical Sciences, Prince of Songkla University. All three interruptins were extracted in the same fraction and further purified using open column chromatography in silica gel and eluted by using ethyl acetate with *n*-hexane (1:4). Thin-layer chromatography was performed to identify the compounds. The purity of the isolated compounds was determined using high-performance liquid chromatography.

### 4.2. Cell Culture and X-ray Irradiation

The MDA-MB-231 and Hs578T human breast cancer cell lines were used as cancer cell models. MCF-10A normal breast cells from the mammary gland and HaCaT human keratinocytes from the epidermis layer of the skin were used as normal cells. The HaCaT cell line was a generous gift from Associate Professor Dr. Sireewan Kaewsuwan, whereas MDA-MB-231, Hs578T, and MCF-10A cells were purchased from American Type Culture Collection (Manassas, VA, USA). MDA-MB-231, Hs578T, and HaCaT cells were cultured in Dulbecco’s modified Eagle’s medium (Gibco, Grand Island, NY, USA) supplemented with 10% fetal bovine serum (Gibco), 100 U/mL of penicillin–streptomycin (Gibco), and 3.7 g/L sodium bicarbonate (Sigma, St. Louis, MO, USA). MCF-10A cells were cultured in Dulbecco’s modified Eagle’s medium: F12 (Gibco) supplemented with 10% heat inactivated fetal bovine serum, 20 ng/mL human epidermal growth factor (Sigma), 250 ng/mL hydrocortisone (Sigma), 10 µg/mL insulin (Sigma), 10 mL penicillin–streptomycin (Gibco), and 2.438 g/L sodium bicarbonate (Sigma). All cells were maintained at 37 °C with 5% CO_2_ in humidified air.

Irradiation was performed using a 6-MV X-ray TrueBeam™ STx linear accelerator (Varian Medical System, Tucson, AZ, USA) with a single dose, irradiation field of 30 × 30 cm, and dose rate of 400 MU/min. The irradiation doses were 0, 1, 2, 5, 10, and 15 Gy based on the doses routinely used in radiotherapy. The measured dose versus the planned dose was determined using a FC65-G dosimeter and Dose1 electrometer (IBA Dosimetry, Schwarzenbruck, Germany) (Figures S2 and S3 in Supplementary Materials). The cells were grown in a 12-well plate for the clonogenic survival assay and 96-well plate for the MTT assay. Cells were treated with 2 µM of interruptin compounds for 1 h before irradiation. The selected concentration and incubation time of interruptin were optimized to minimize the cytotoxicity of the compound. All groups of cells were trypsinized and transferred into 15-mL tubes. Cells in the tubes were gently mixed before irradiation in each session to prevent them from precipitating during irradiation. Amifostine (Sigma) and resveratrol (Sigma) were used as positive controls of synthetic and natural radioprotective agents, respectively. The tubes were filled with the medium and irradiated horizontally in the upright position with a solid water phantom (tissue-equivalent material) in front of and behind the tubes to ensure electronic equilibrium.

### 4.3. DCFH-DA Assay

A previous study [14] showed that coumarin compounds, including interruptin compounds, have antioxidant activity. We investigated the antioxidant activity of interruptins in HaCaT cells by measuring intracellular ROS in a DCFH-DA assay using 2′,7′-dichlorofluorescin diacetate (DCFH-DA). DCFH-DA is converted into DCFH by esterase enzyme in the cell. The oxidation of DCFH into fluorescent DCF due to the presence of ROS was measured [49]. The cells were cultured at 3.5 × 10^4^ cells/well in 96-well plates for 24 h and treated with a final concentration of 1.5 mM H_2_O_2_. The cells were incubated at 37 °C for 24 h in a humidified atmosphere with 5% CO_2_ with all compounds and Trolox as the positive control at final concentrations of 2 and 500 µM, respectively. The cells were supplemented with DCFH-DA solution at a final concentration of 10 µM. Excitation and emission wavelengths of 485 and 530 nm were used to measure the fluorescence intensity of fluorescent DCF.

### 4.4. MTT Assay

The MTT (3-(4,5-dimethylthiazol-2-yl)-2,5-diphenyltetrazolium bromide) assay is based on the conversion of yellow-colored tetrazolium MTT into purple-colored formazan crystals by the mitochondrial reductase enzyme in living cells. The amount of formazan crystals is proportional to the metabolic activity in living cells. The MTT assay was performed to detect the short-term response of cells after chemical or radiation exposure. Irradiated MDA-MB-231, Hs578T, HaCaT, and MCF-10A cells were seeded at 10,000–20,000 cells per well, depending on the cell type, into a 96-well plate. The MTT solution was prepared in phosphate-buffered saline (PBS) at concentrations of 0.5 mg/mL, and 100 µL of this solution was added to each well and incubated at 37 °C for 30–45 min. The remaining MTT solution was removed, and then, 100 µL of 100% dimethyl sulfoxide was added to each well to dissolve the formazan crystals at 37 °C for 30–45 min; the incubating time differed for the different cell types [50]. The absorbance of each well was measured at 570 nm, with the reference of absorbance at 650 nm using a microplate reader.

### 4.5. Clonogenic Cell Survival Assay

Clonogenic cell survival assays are commonly used to investigate the long-term survival of irradiated cells. The most common mechanisms described for cell death are the loss of reproductive integrity and inability to proliferate. Therefore, cells showing these features were considered as dead and unable to divide and produce large colonies. Cells showing the capacity to divide, proliferate, and produce a large colony are considered clonogenic. After irradiation, the cells were added to 12-well plates in triplicate and cultured for up to 10–14 days, depending on the cell types, until colonies formed. Colonies were washed with PBS and fixed with 4% paraformaldehyde for 15 min at room temperature. Visible colonies consisting of at least 50 cells were stained with 0.2% crystal violet (Sigma) and air-dried at room temperature for 2 to 3 days [32,33]. Alternatively, the colonies were quantified using a colorimetric method. Acetic acid (1%) was added to solubilize the crystal violet, and the optical density was measured at 492 nm using a microplate reader [51,52,53,54,55].

### 4.6. SOD Activity Assay

Ionizing radiation kills cells by increasing mitochondrial ROS. Cells contain antioxidants enzymes to prevent or repair the damage caused by ROS. The SOD activity assay was used to measure the antioxidant enzyme activity of SOD [56], using an SOD activity assay kit (Trevigen, Gaithersburg, MD, USA). Superoxide radical ions generate from the conversion of xanthine to uric acid, and H_2_O_2_ by xanthine oxidase converts WST-1 to WST-1 formazan. WST-1 formazan absorbs light at 450 nm. SOD reduces superoxide ion concentrations, thereby lowering the rate of WST-1 formazan formation. The extent of reduction in the WST-1 formazan represents of SOD activity level. The protein extract was used with the SOD activity assay kit. The pellets were isolated at 24 h after irradiation, and the reactions were initiated by adding xanthine solution at 25 µL/well in a 96-well plate. The plate was immediately transferred to a plate reader, and absorbance was measured at 450 nm every minute for 10 min at room temperature. The total SOD activity was calculated using the following equation:

$$\%\;\mathrm{Inhibition}=\frac{\left(\mathrm{slope}\;\mathrm{of}\;1X\;\mathrm{SOD}\;\mathrm{buffer}\;\mathrm{control}\;-\;\mathrm{slope}\;\mathrm{of}\;\mathrm{sample}\right)\;}{\mathrm{slope}\;\mathrm{of}\;1X\;\mathrm{SOD}\;\mathrm{buffer}\;\mathrm{control}}\times 100$$

### 4.7. γH2AX Assay

H2AX molecules appear within minutes after irradiation. First, H2AX phosphorylation occurs in the chromatin surrounding the DNA DSB site. Next, hundreds of γH2AX molecules surround this site to form a focus that functions to open the chromatin structure and interact with other molecules to perform DNA damage repair [57]. After irradiation, cells grown on slides were fixed with 4% paraformaldehyde at 1, 3, 6, and 24 h, followed by treatment with 0.2% Triton X-100 in PBS and blocking with 5% fetal bovine serum (9.5 mL 1X PBS, 0.5 mL fetal bovine serum, and 30 µL Triton X-100) for 40 min at room temperature. The cells were incubated at 4 °C with the primary antibody phospho-histone H2AX (Ser139) (20E3) (Cell Signaling Technology, Danvers, MA, USA) at 1:800 diluted with the dilution buffer (10 mL 1X PBS, 30 µL Triton X-100, and 0.1 g bovine serum albumin) overnight. The cells were washed with PBS, and the secondary antibody Alexa Fluor 488 (1:800) was added for 45 min at room temperature in the dark (covered in foil). The cells were stained with 1 µg/mL DAPI for 10 min before mounting with the mounting medium. Images were collected with a fluorescence microscope. Under each condition, images of at least 100 cells were captured and used to quantitatively analyze the γH2AX foci [58]. ImageJ software (NIH, Bethesda, MD, USA) was used to count the γH2AX foci. First, the number of nuclei in a field was counted. The threshold of the image was set to an optimal value, creating a single region in each nucleus. The γH2AX foci were counted using the “Find Maxima” tab menu.

### 4.8. MN Formation Assay

The induction of small membrane-bound DNA fragments or MN indicates DNA DSB and chromatin breaks. An MN formation assay or cytokinesis block MN assay can be used to measure DNA damage and provide information on cell cycle progression. MN are formed during mitosis when chromatin fragments are not distributed into daughter nuclei. The cytokinesis block method has been developed to detect whether radiation induces MN formation in interphase cells. The actin polymerization inhibitor cytochalasin B was added, the cells have undergone first division as binucleates for the identification of MN. After irradiation, the cells were seeded at 500 cells/well into a 24-well plate. At 24 h after irradiation, the cells were treated with 3 μg/mL cytochalasin B (Sigma) to inhibit cytokinesis and then incubated for a further 24 h. The cells were harvested by trypsinization and centrifuged at 1500 rpm for 5 min. The cells were fixed with cold 100% methanol for 15 min, suspended in cold methanol: glacial acetic acid (3:1 *v*/*v*), dropped onto cold glass slides, and dried at room temperature. The slides were stained with a Giemsa solution (Sigma) (1:20 in distilled water) for 15 min and analyzed under a light microscope at 100× magnification. Images of 200 binucleated cells were captured and used for quantitative analyses of MN. Scoring was performed for MN with a diameter that was less than 1/3 of that of the main nucleus [59]. The example of captured images of the cells scored in the micronuclei formation assay are shown in Figure S4 in Supplementary Materials.

### 4.9. Western Blot Analysis

Western blot assay was used to monitor proteins related to the DNA damage response after cell irradiation [60]. The proteins were separated using gel electrophoresis. Cell pellets were collected after irradiation at 1, 3, 6, and 24 h. Total proteins were extracted using 20–30 µL of RIPA buffer, depending on the amount of cell pellets. Proteins (50 µg) were separated by electrophoresis on 12% sodium dodecyl sulfate polyacrylamide gels in running buffer (25 mM Tris-base, 190 mM glycine, and 0.1% SDS). An equal amount of protein was loaded in each lane. These proteins were transferred onto nitrocellulose membranes in transfer or blotting buffer, and the membrane was blocked with 5% skim milk or 5% bovine serum albumin (for phosphorylation proteins) in TTBS (48 mM Tris-base, 154 mM NaCl, and 0.1% Tween-20) for 1 h and washed 3 times with washing buffer (1% skim milk or 1% BSA in TTBS). The membranes were incubated with primary antibody at 4 °C overnight. Western blotting was performed using the following antibodies: Chk1 (1:500), p-Chk1 (S345) (1:500), γH2AX (S139) (1:1000), p53 (1:1000), p-p53 (S15) (1:1000), Bcl-2 (1:1000), Bax (1:1000), and β-actin (1:1,000). All antibodies were purchased from Cell Signaling Technology. The membranes were washed 3 times with washing buffer and then incubated with anti-rabbit IgG (horseradish peroxidase) or anti-mouse IgG (horseradish peroxidase) for 1 h at room temperature. All membranes were detected with a chemiluminescence detection kit, SuperSignal West Femto chemiluminescence substrate and SuperSignal West Dura chemiluminescence substrate (Pierce, Rockford, IL, USA).

### 4.10. Statistical Analysis

All experiments were performed in triplicate, and the data are shown as the mean standard deviation from three independent repeats. Statistical significance was tested using R program software (The R Project for Statistical Computing, Vienna, Austria). The data were tested for a normal distribution prior to statistical analysis using the Shapiro–Wilk normality test. To optimize the irradiation setup, significant differences between two groups were evaluated using a paired *t*-test. Significant differences in the data from the DCFH-DA, MTT, clonogenic cell survival, SOD activity, γH2AX, MN formation, and Western blot assays among more than two groups were evaluated using Kruskal–Wallis with Dunn’s test. Differences were considered significant when *p* < 0.05.

## Data Availability

Not applicable.

## Supplementary Materials

The following supporting information can be downloaded at https://www.mdpi.com/article/10.3390/molecules27103298/s1: Figure S1: Radioprotective activity pathways. Figure S2: The dose measurement setup. Figure S3: The irradiation setup for the cell culture. Figure S4: The example of captured images of the cells scored in the micronuclei formation assay. Figure S5: γH2AX protein expressions. Figure S6: p-p53/p53 protein expressions. Figure S7 p-chk1/chk1 protein expressions. Figure S8: Bcl-2 protein expressions. Figure S9. Bax protein expressions.
