# Supplementary material for: Interruptin C, a Radioprotective Agent, Derived from Cyclosorus terminans Protect Normal Breast MCF-10A and Human Keratinocyte HaCaT Cells against Radiation-Induced Damage
Source: Molecules. 2022 May 20;27(10):3298. doi: 10.3390/molecules27103298 (PMC9142933; doi:10.3390/molecules27103298)
Supplement: Supplementary file 1 [file molecules-27-03298-s001.zip › molecules-1638881-supplementary.pdf]

Interruptin C, a radioprotective agent, derived from *Cyclosorus terminans* protect normal breast MCF-10A and human keratinocyte HaCaT cells against radiation-induced damage

Nipha Chumsuwan [1, 2], Pasarat Khongkow [1], Sireewan Kaewsuwan [3], and Kanyanatt Kanokwiroon [1,*]

[1] Department of Biomedical Sciences and Biomedical Engineering, Faculty of Medicine, Prince of Songkla University, Hat Yai, Songkhla 90110, Thailand; noknipha@hotmail.com (N.C.); k.pasarat@gmail.com (P.K.)
[2] Department of Radiology, Faculty of Medicine, Prince of Songkla University, Hat Yai, Songkhla 90110, Thailand
[3] Department of Pharmacognosy and Pharmaceutical Botany, Faculty of Pharmaceutical Sciences, Prince of Songkla University, Hat Yai, Songkhla 90110, Thailand; songsri.k@psu.ac.th (S.K.)
* Correspondence: kanyanatt.k@psu.ac.th (K.K.); Tel.: +6674451185, +66816983448

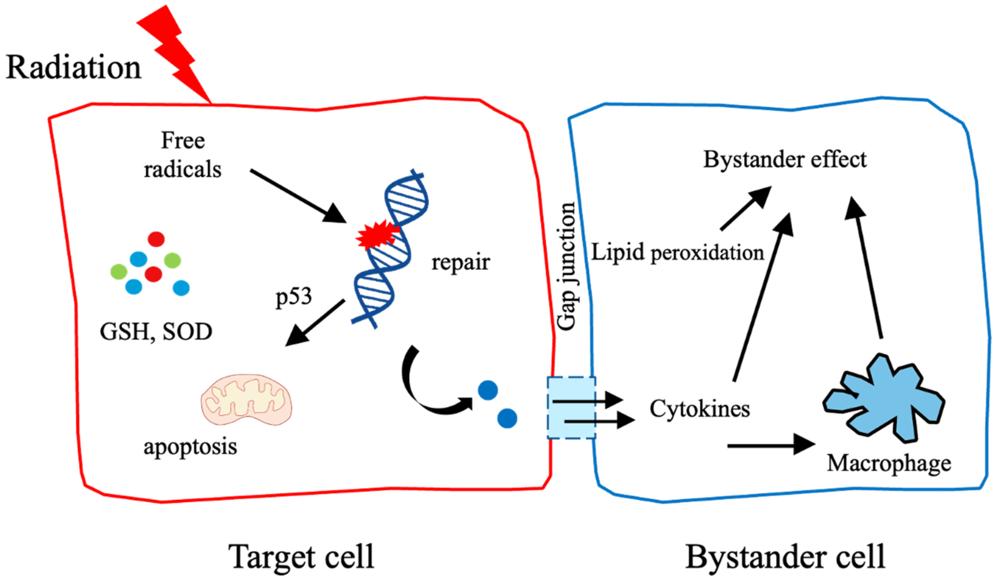

Supplementary Materials Figure S1. Radioprotective activity pathways. The radiation-induced damage mechanisms provide strategies to manage the radiation injury by using radioprotectors. They may be reducing the free radical formation by chain reaction inhibition and activate cellular protective enzyme by enhancing the activity level of superoxide dismutase (SOD) and glutathione enzymes. Furthermore, radioprotectors are helpful to reduce radiation injury by DNA repair enhancement, stimulating immune response, and decreasing bystander effect.

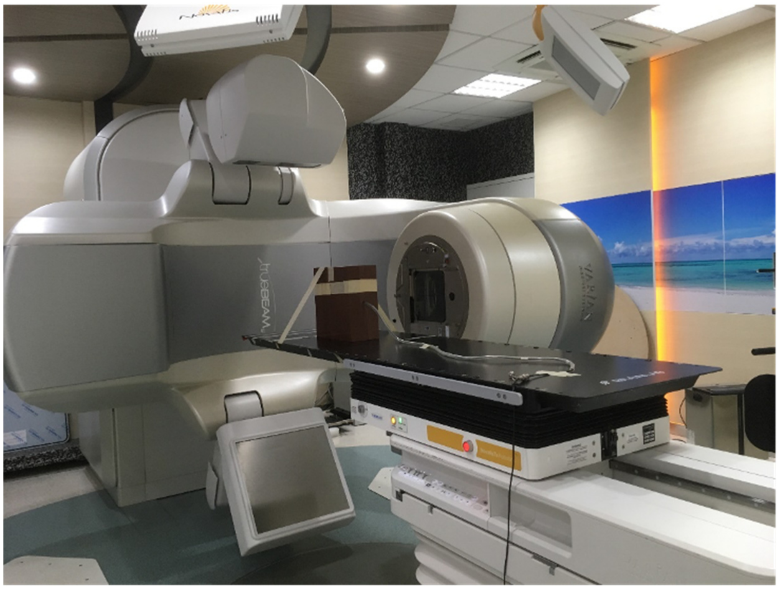

Supplementary Materials Figure S2. The dose measurement setup with dosimeter was done using TrueBeam™ STx Linac irradiation machine.

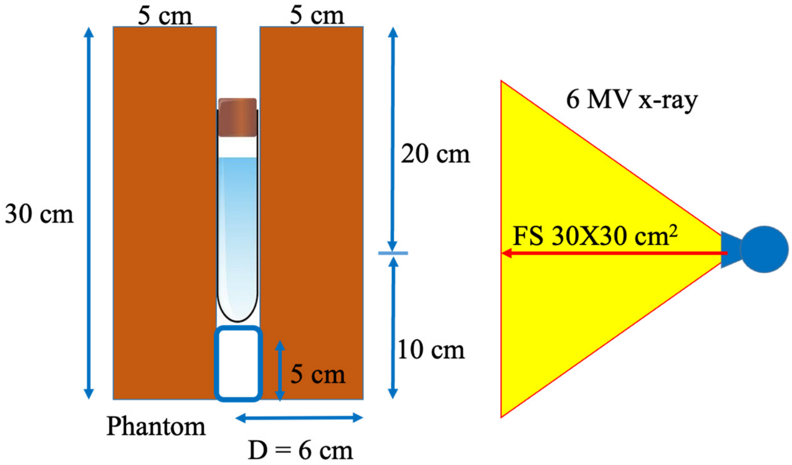

Supplementary Materials Figure S3. The irradiation setup for cell culture using 6 MV x-ray with field size (FS) 30 X 30 cm^2, dose rate 400 MU/minute, depth 6 cm.

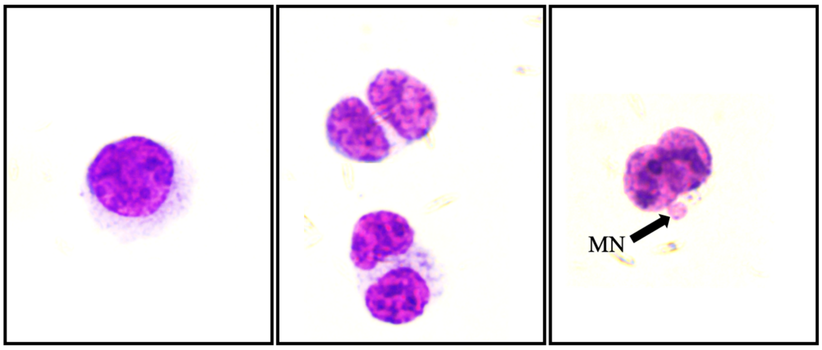

Supplementary Materials Figure S4. The example of captured images of the cells scored in micronuclei formation assay, momonucleated cell (left panel), binucleated cell (middle panel) and binucleated cells with micronuclei (right panel).

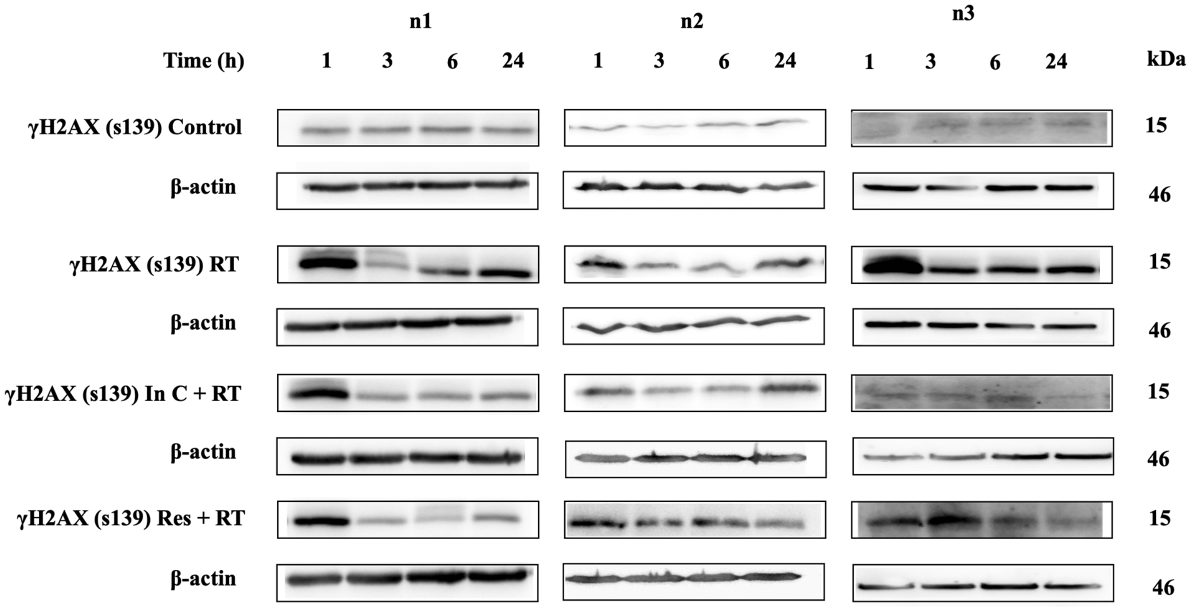

Supplementary Materials Figure S5. γH2AX protein expressions were evaluated using Western blotting under different condition including cell without irradiation, irradiated cells, interruptin C pretreatment and resveratrol pretreatment at 1, 3, 6 and 24 hours after irradiation exposure of HaCaT cells. γH2AX proteins were depicted along with their β-actin. The results were performed in three independent experiments (n1-n3). The experiment n1-n3 used for calculation of band

intensity or relative expression from γH2AX protein and β-actin. The full original Western blot images was shown in this revision as follows: γH2AX (s139) Control, γH2AX (s139) RT, γH2AX (s139) In C + RT and γH2AX (s139) Res + RT. In full original blot, Black outer square; border of full images, Red inner square; indicated protein bands.

γH2AX (s139) Control

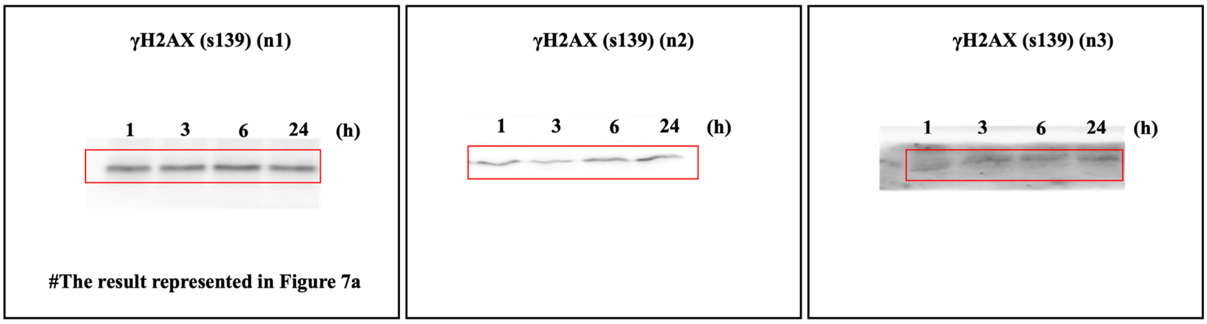

β-actin Control

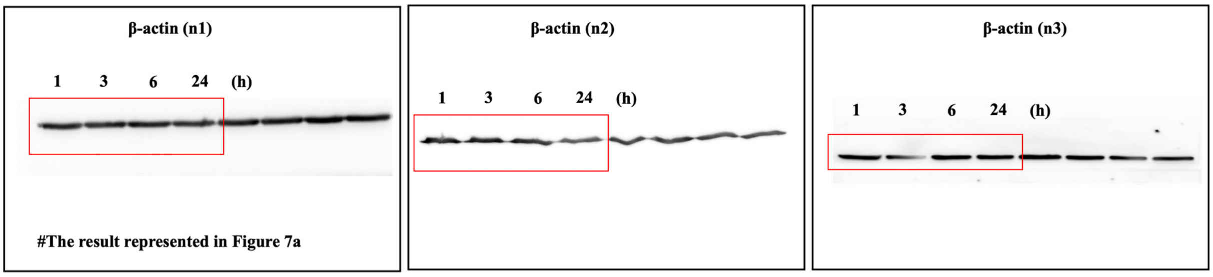

γH2AX (s139) RT

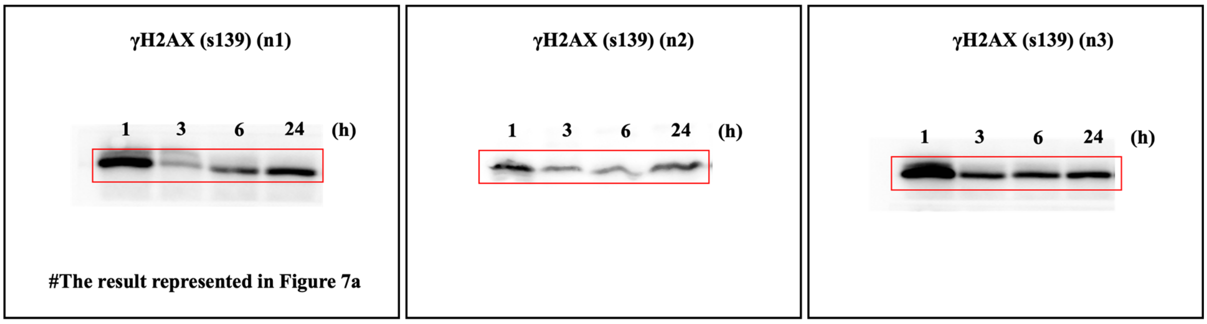

β-actin RT

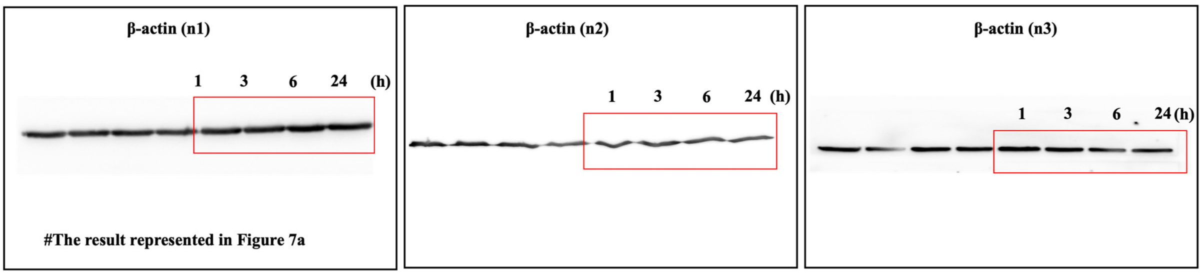

γH2AX (s139) In C + RT

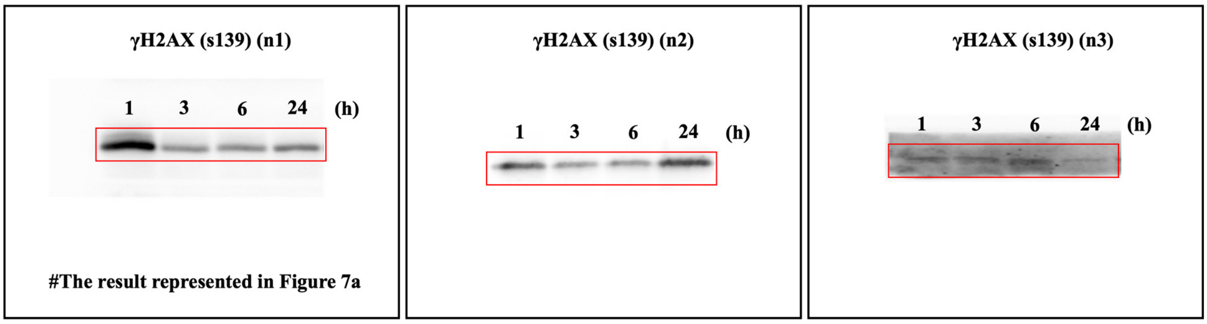

β-actin In C + RT

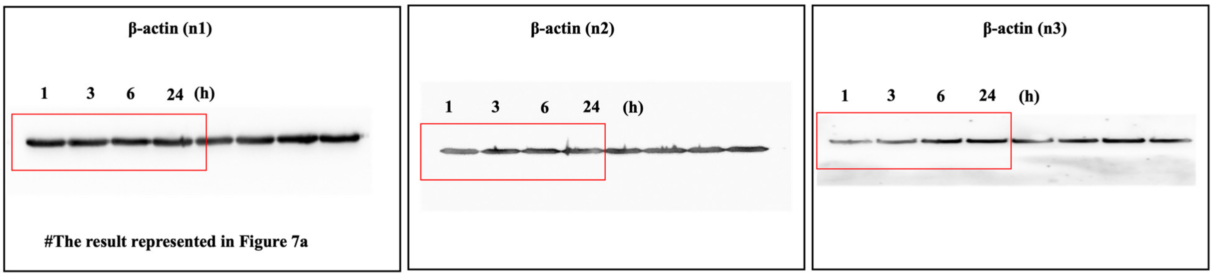

γH2AX (s139) Res + RT

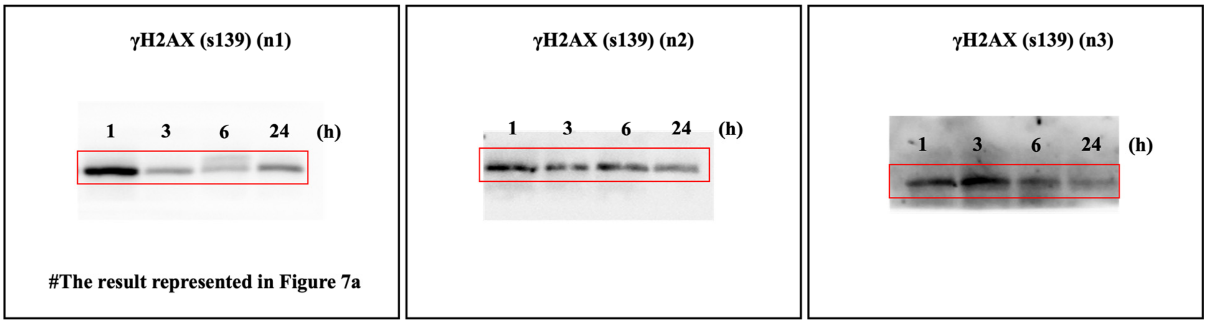

β-actin Res + RT

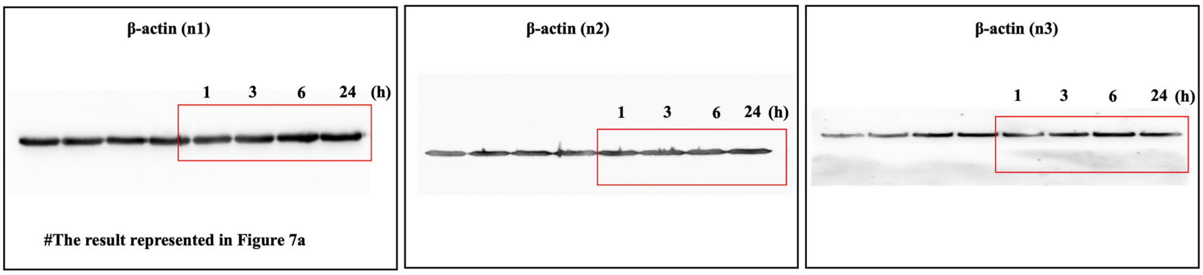

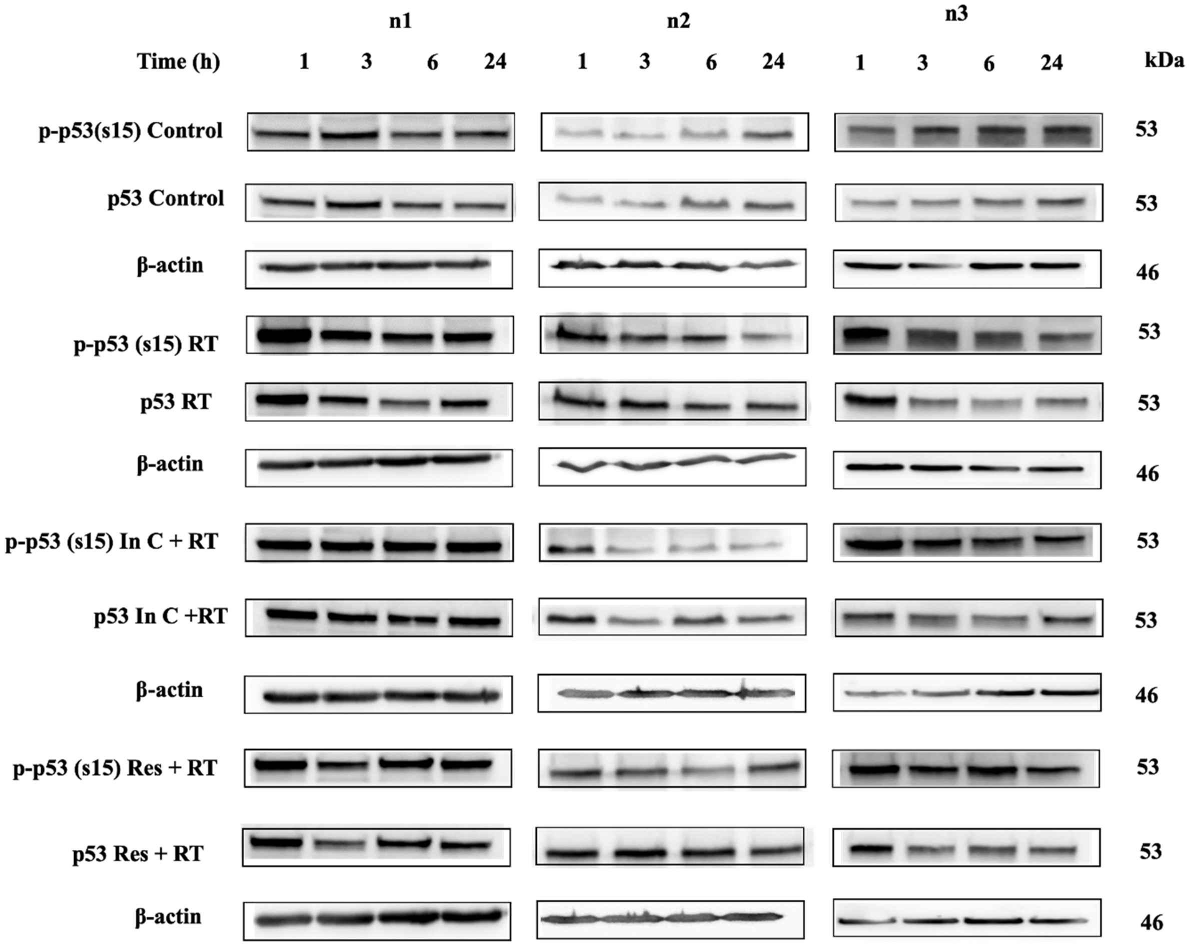

Supplementary Materials Figure S6. p-p53/p53 protein expressions were evaluated using Western blotting under different condition including cell without irradiation, irradiated cells, interruptin C pretreatment and resveratrol pretreatment at 1, 3, 6 and 24 hours after irradiation exposure of HaCaT cells. These proteins were depicted along with their β-actin. The results were

performed in three independent experiments (n1-n3). The experiment n1-n3 used for calculation of band intensity or relative expression from indicated protein and β-actin. The full original Western blot images was shown in this revision as follows: p-p53 (s15) Control, p53 Control, p-p53 (s15) RT, p53 RT, p-p53 (s15) In C + RT, p53 In C + RT, p-p53 (s15) Res + RT, p53 Res + RT. In full original blot, Black outer square; border of full images, Red inner square; indicated protein bands.

p-p53 (s15) Control

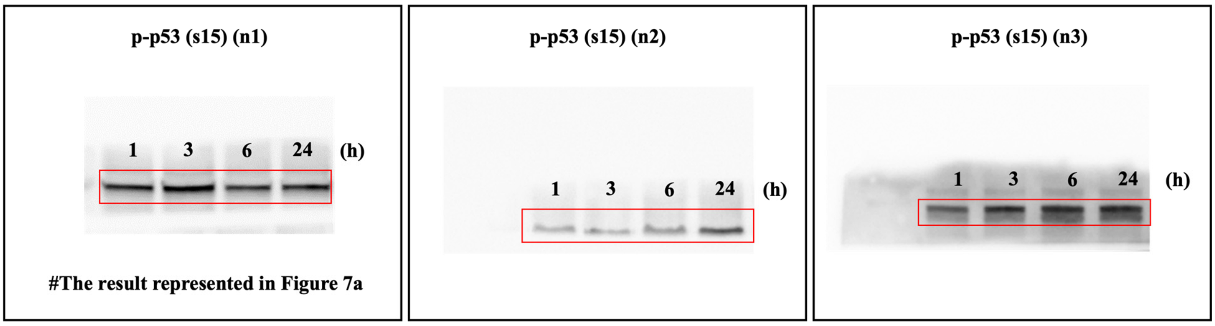

p53 Control

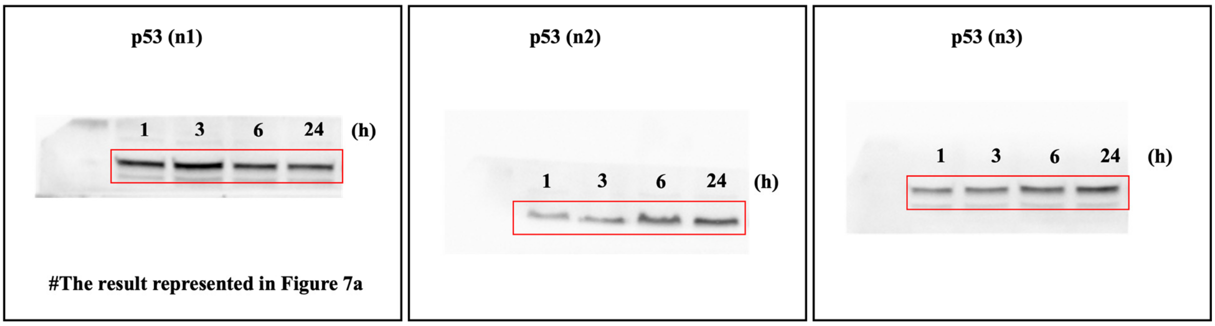

β-actin Control

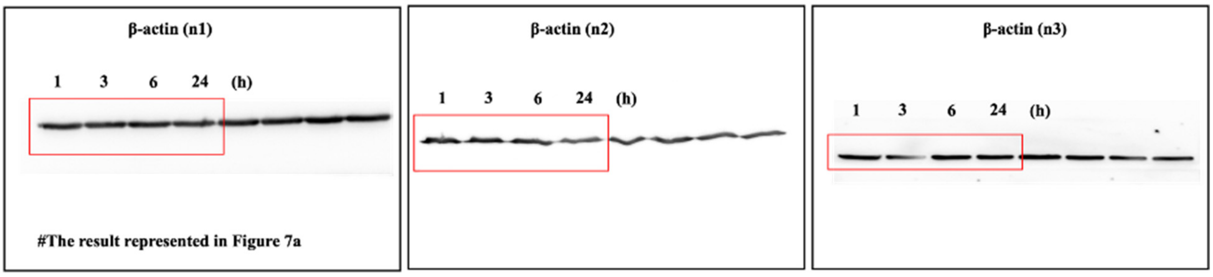

p-p53 (s15) RT

p-p53 (s15) (n1)

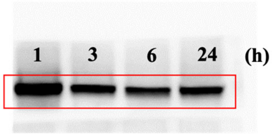

#The result represented in Figure 7a

p-p53 (s15) (n2)

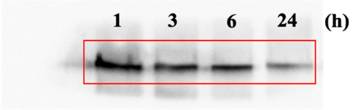

p-p53 (s15) (n3)

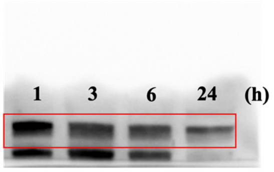

p53 RT

p53 (n1)

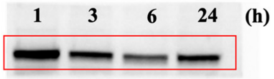

#The result represented in Figure 7a

p53 (n2)

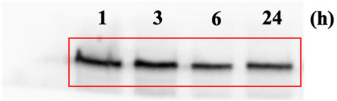

p53 (n3)

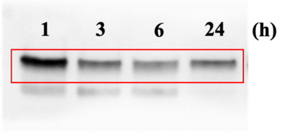

β-actin RT

β-actin (n1)

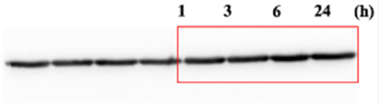

#The result represented in Figure 7a

β-actin (n2)

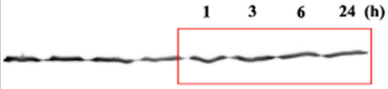

β-actin (n3)

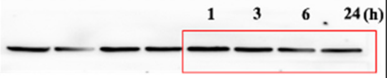

p-p53 (s15) In C + RT

p-p53 (s15) (n1)

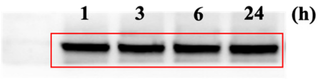

#The result represented in Figure 7a

p-p53 (s15) (n2)

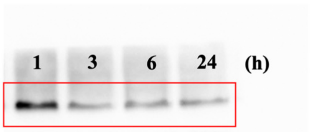

p-p53 (s15) (n3)

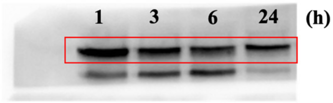

p53 In C + RT

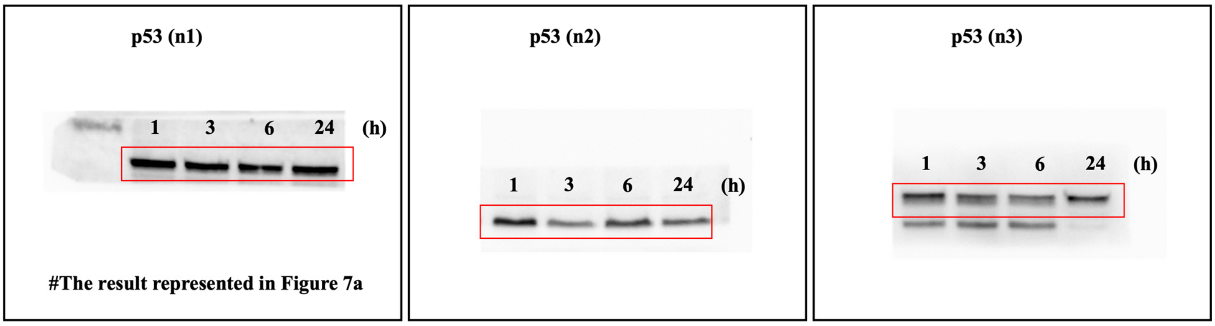

β-actin In C + RT

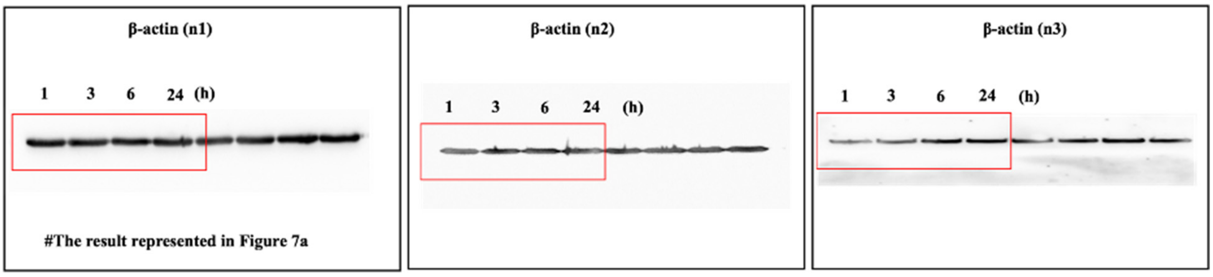

p-p53 (s15) Res + RT

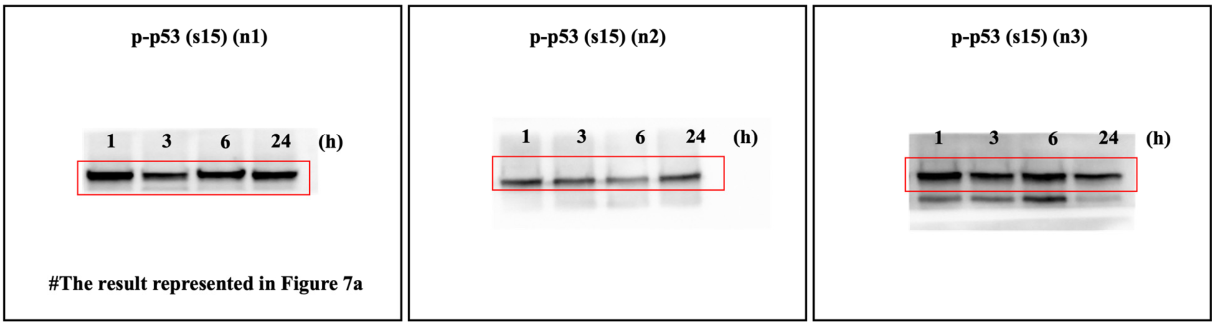

p53 Res+ RT

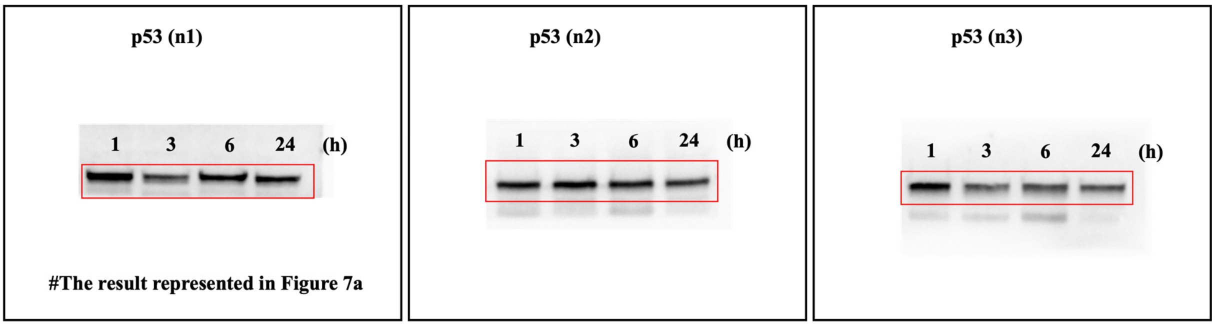

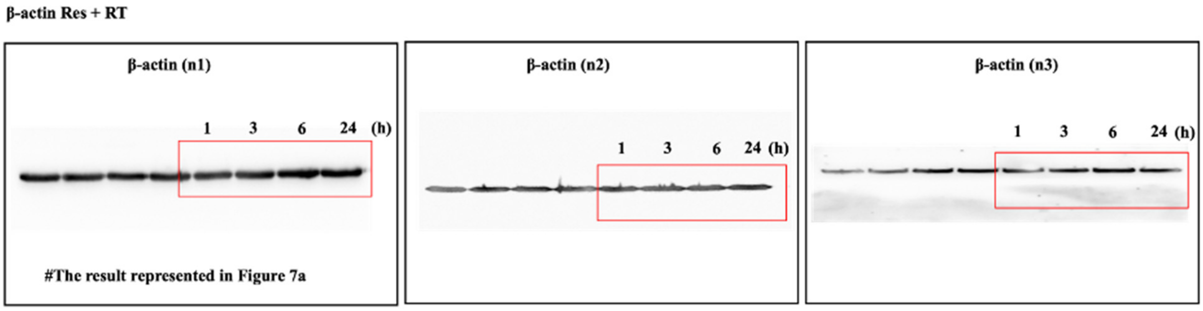

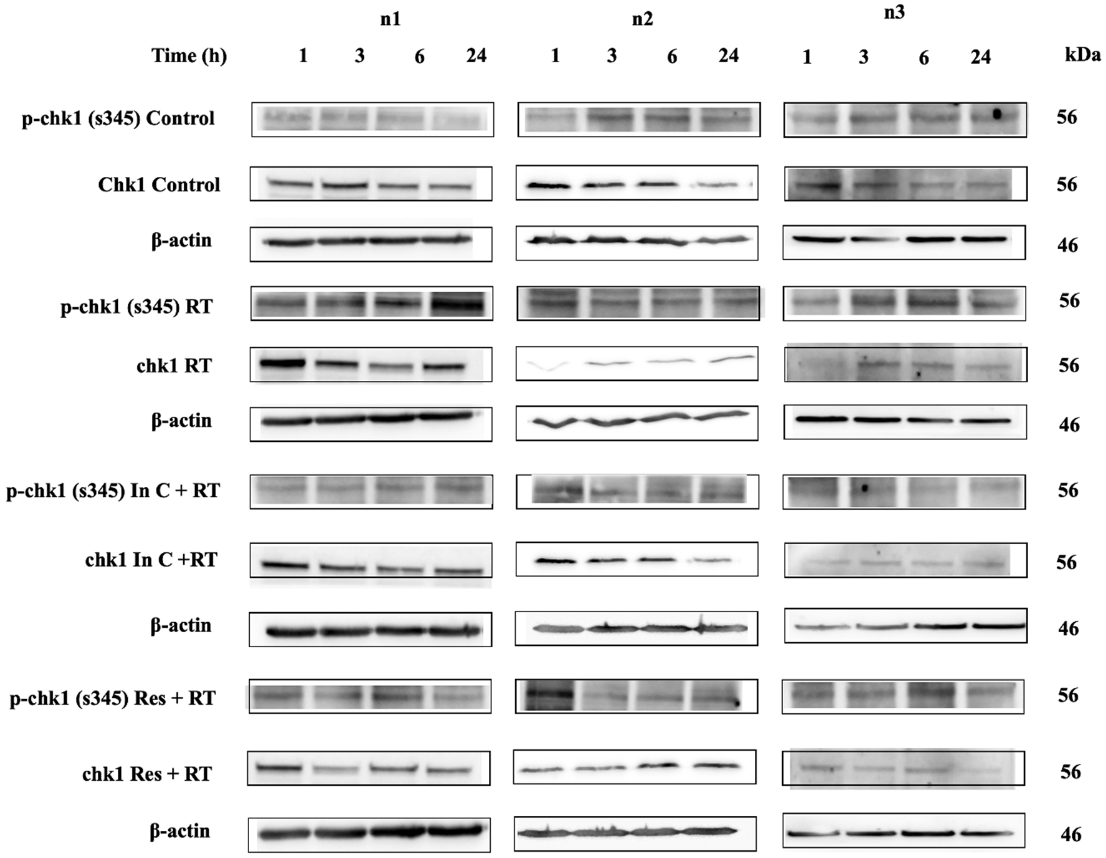

Supplementary Materials Figure S7. p-chk1/chk1 protein expressions were evaluated using Western blotting under different condition including cell without irradiation, irradiated cells, interruptin C pretreatment and resveratrol pretreatment at 1, 3, 6 and 24 hours after irradiation exposure of HaCaT cells. These proteins were depicted along with their β-actin. The results were performed in three independent experiments (n1-n3). The experiment n1-n3 used for calculation

of band intensity or relative expression from indicated protein and β-actin. The full original Western blot images was shown in this revision as follows: p-chk1 (s345) Control, chk1 Control, p-chk1 (s345) RT, chk1 RT, p-chk1 (s345) In C + RT, chk1 In C + RT, p-chk1 (s345) Res + RT, chk1 Res + RT. In full original blot, Black outer square; border of full images, Red inner square; indicated protein bands.

p-chk1 (s345) Control

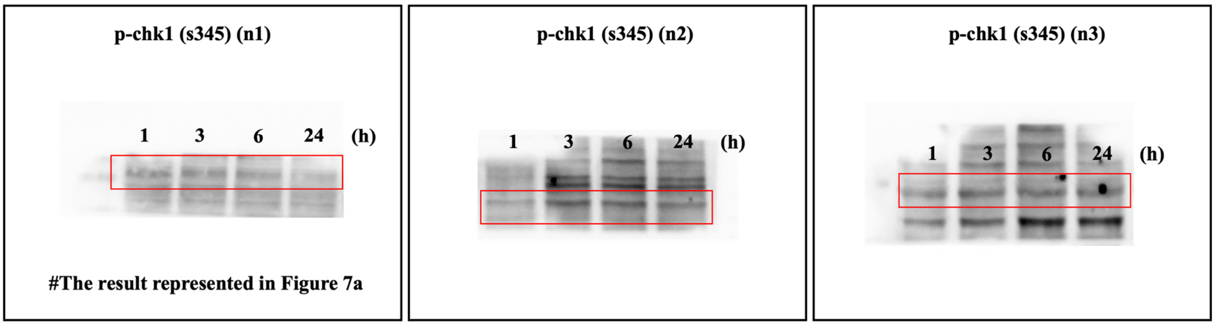

chk1 Control

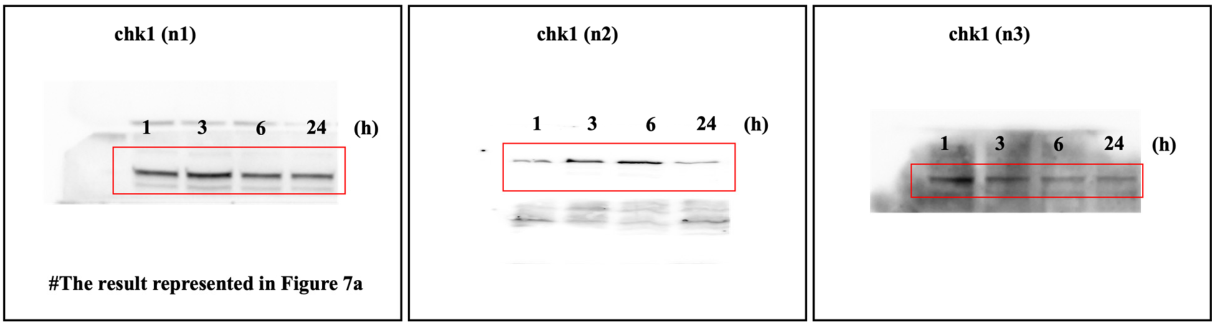

β-actin Control

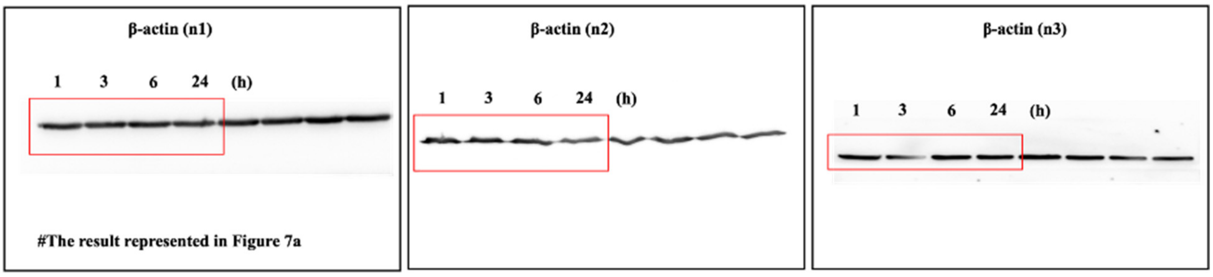

p-chk1 (s345) RT

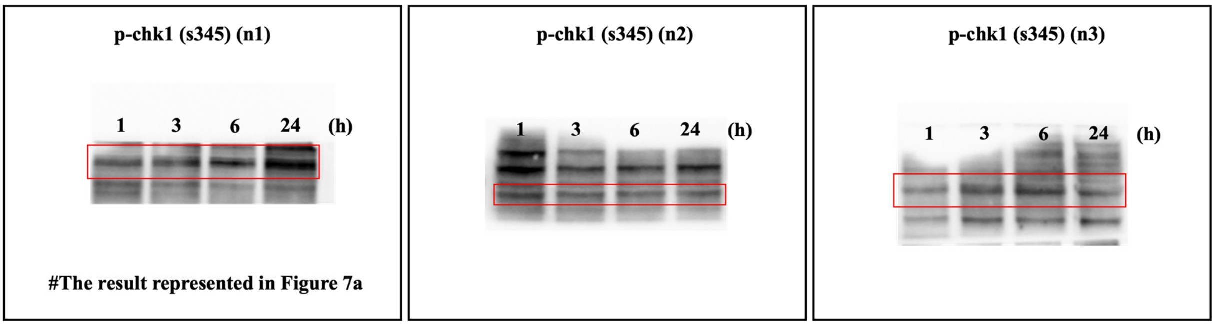

chk1 RT

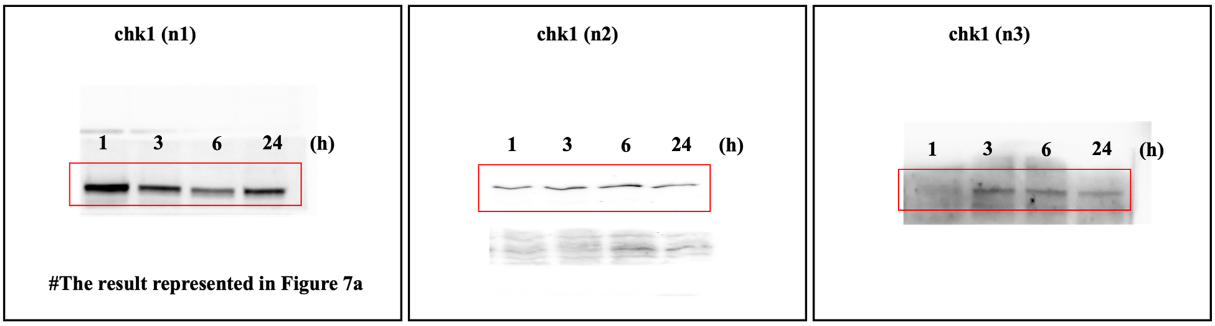

β-actin RT

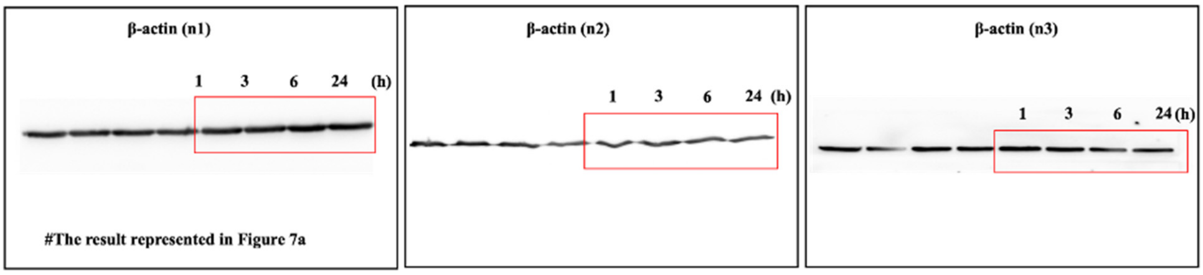

p-chk1 (s345) In C + RT

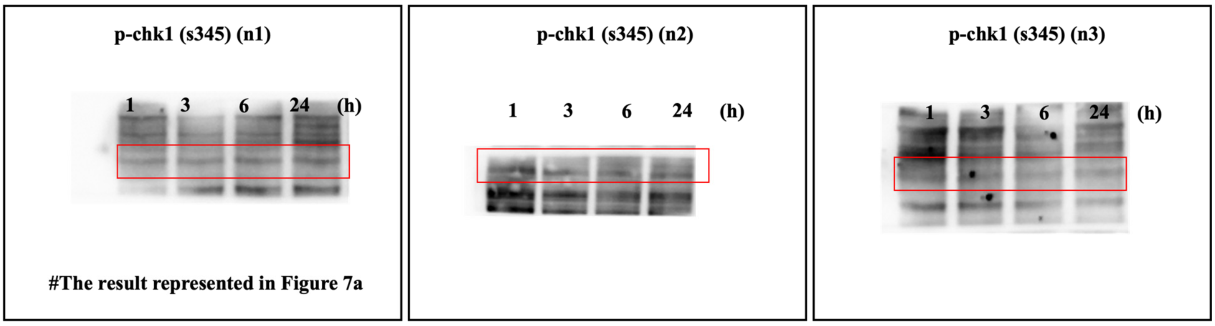

chk1 In C + RT

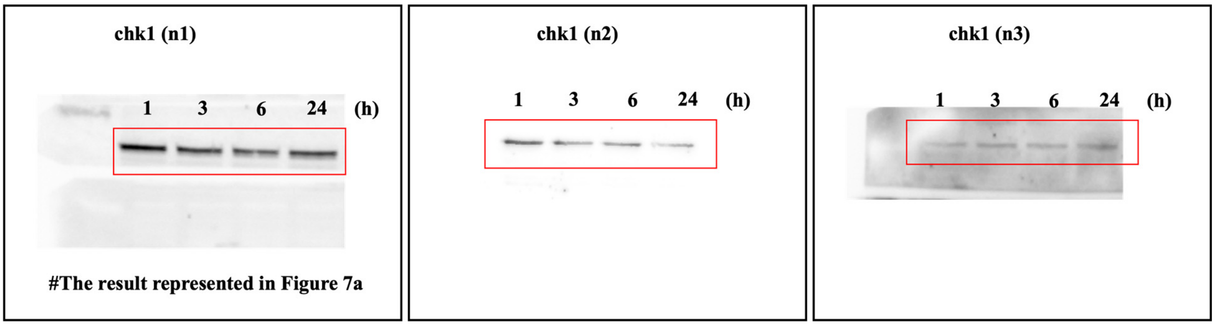

β-actin In C + RT

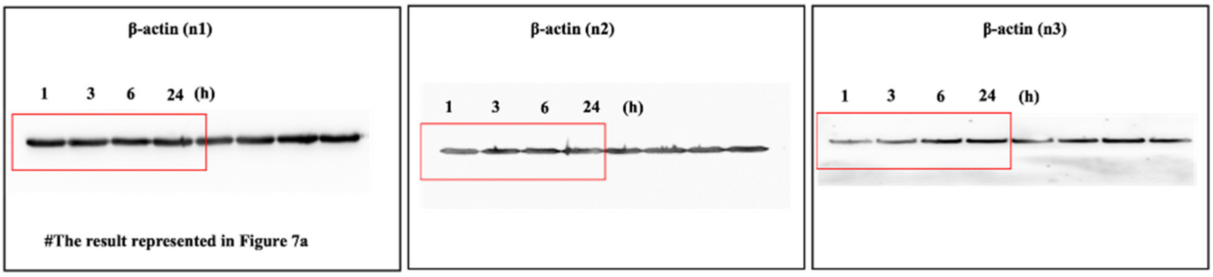

p-chk1 (s345) Res + RT

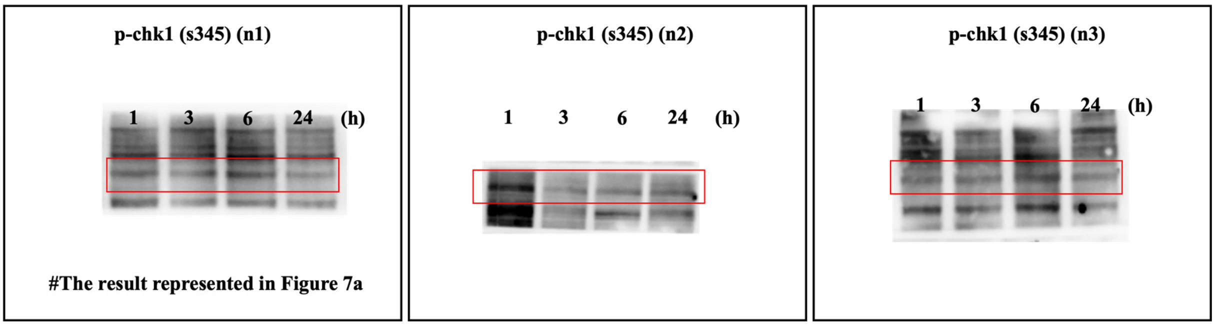

chk1 Res + RT

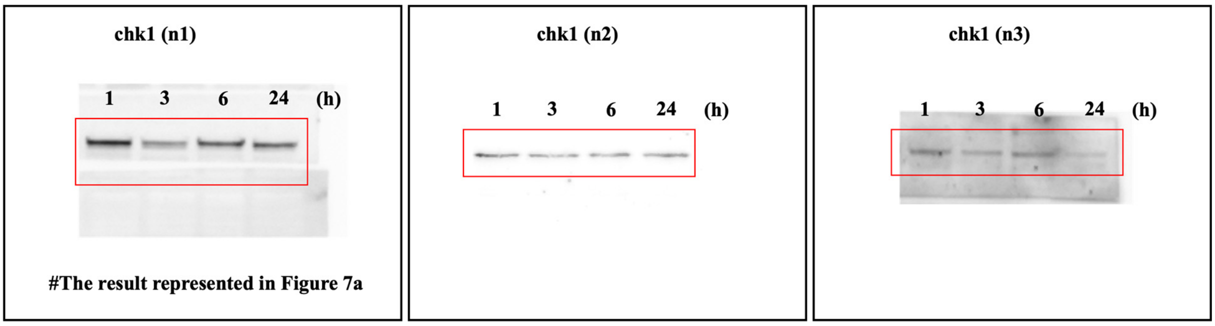

β-actin Res + RT

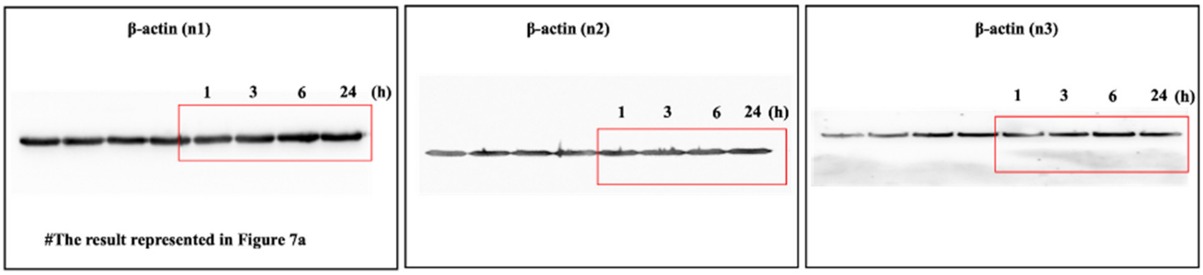

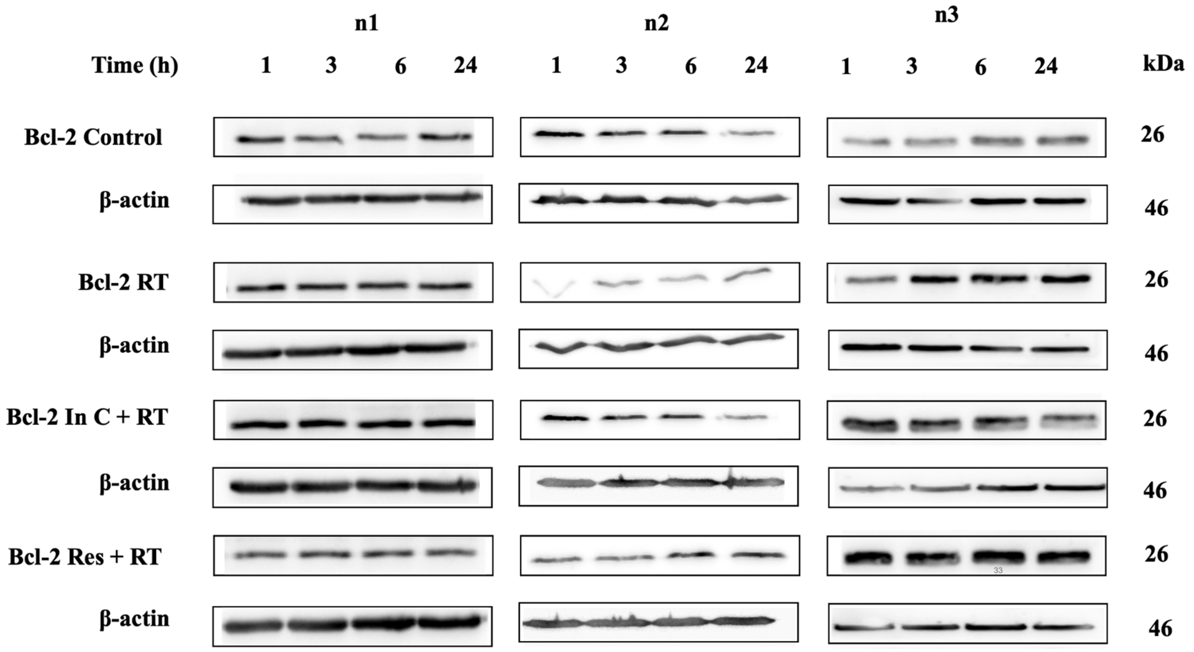

Supplementary Materials Figure S8. Bcl-2 protein expressions were evaluated using Western blotting under different condition including cell without irradiation, irradiated cells, interruptin C pretreatment and resveratrol pretreatment at 1, 3, 6 and 24 hours after irradiation exposure of HaCaT cells. Bcl-2 proteins were depicted along with their β-actin. The results were performed in three independent experiments (n1-n3). The experiment n1-n3 used for calculation of band intensity or relative expression from Bcl-2 protein and β-actin. The full original Western blot images was shown in this revision as follows: Bcl-2 Control, Bcl-2 RT, Bcl-2 In C + RT and

Bcl-2 Res + RT. In full original blot, Black outer square; border of full images, Red inner square; indicated protein bands.

Bcl-2 Control

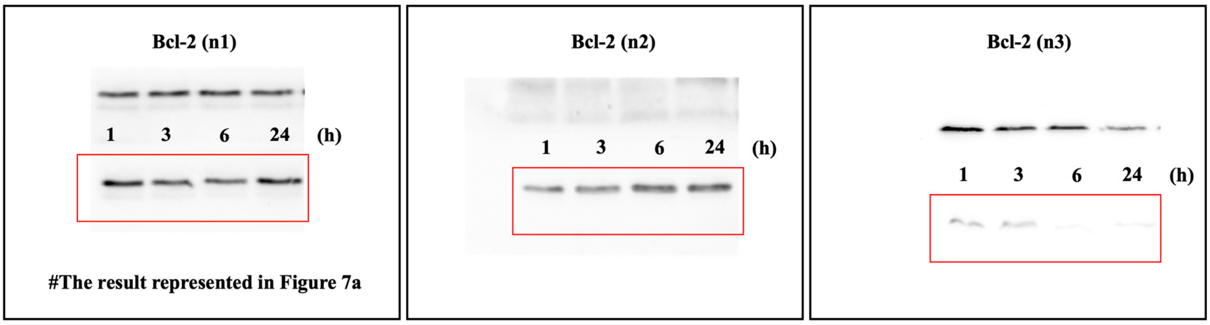

β-actin Control

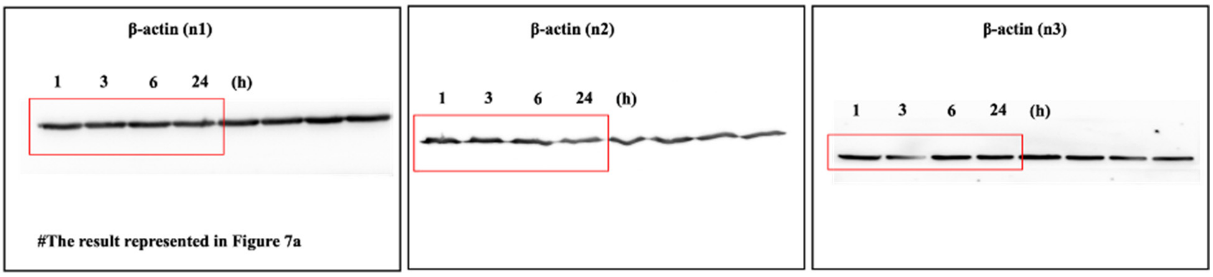

Bcl-2 RT

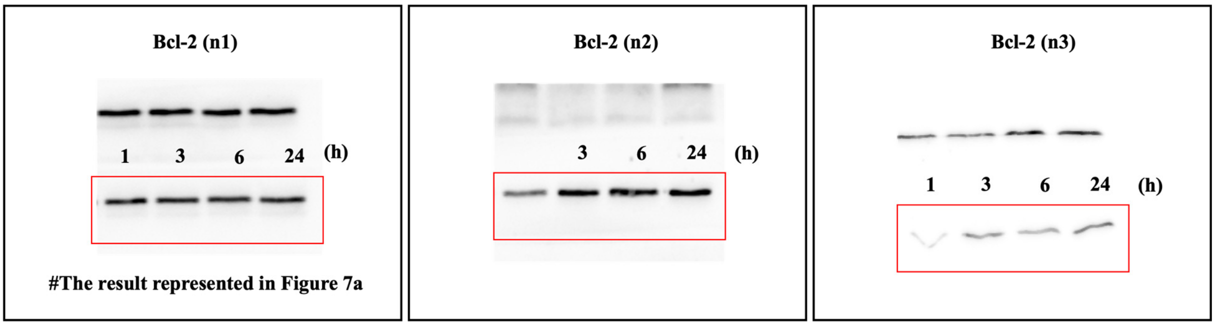

β-actin RT

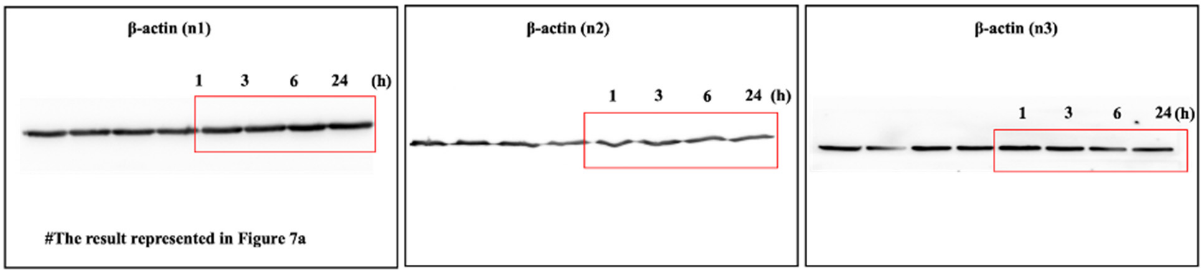

Bcl-2 In C + RT

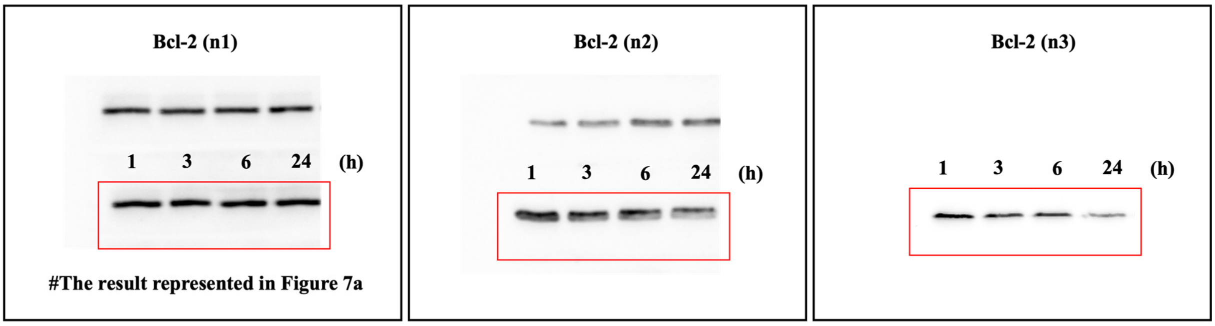

β-actin In C + RT

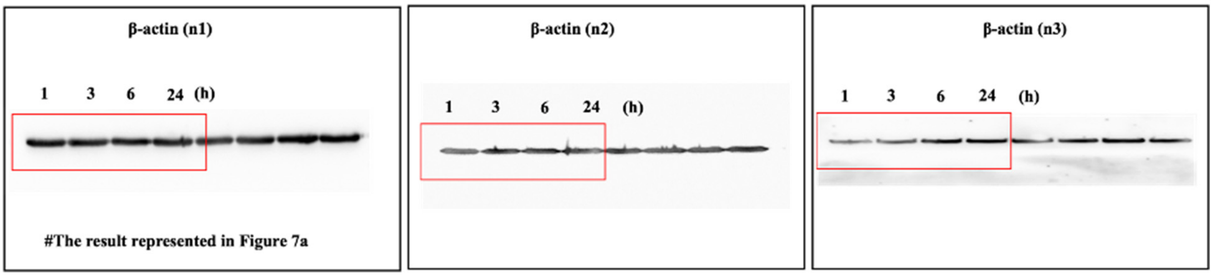

Bcl-2 Res + RT

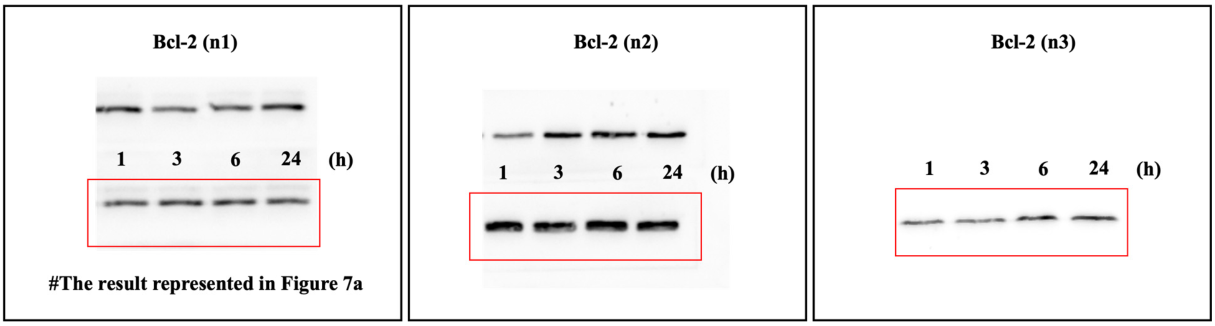

β-actin Res + RT

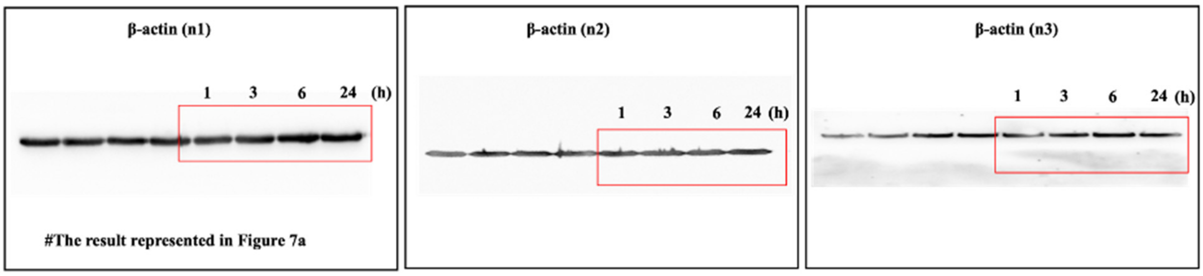

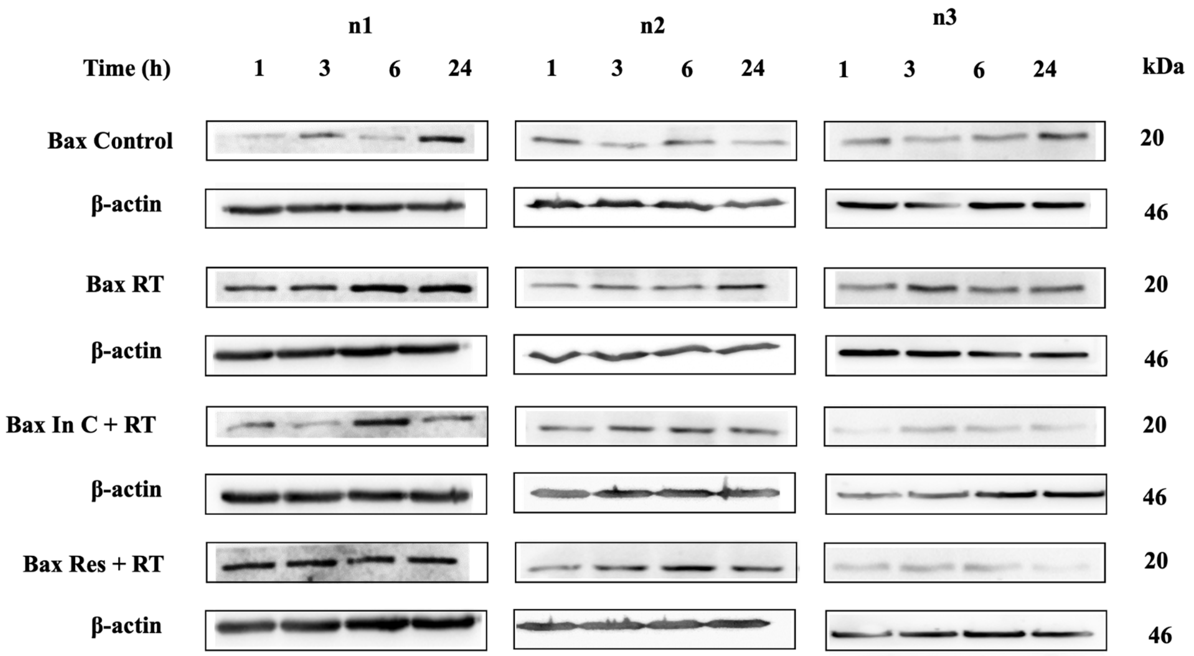

Supplementary Materials Figure S9. Bax protein expressions were evaluated using Western blotting under different condition including cell without irradiation, irradiated cells, interruptin C pretreatment and resveratrol pretreatment at 1, 3, 6 and 24 hours after irradiation exposure of HaCaT cells. Bax proteins were depicted along with their β-actin. The results were performed in three independent experiments (n1-n3). The experiment n1-n3 used for calculation of band intensity or relative expression from Bax protein and β-actin. The full original Western blot images was shown in this revision as follows: Bax Control, Bax RT, Bax In C + RT and Bax Res + RT. In full original blot, Black outer square; border of full images, Red inner square; indicated protein bands.
